# Assessment of *Fusarium*-Damaged Kernels in Common Wheat in Romania in the Years 2015 and 2016 with Extreme Weather Events

**DOI:** 10.3390/toxins14050326

**Published:** 2022-05-04

**Authors:** Valeria Gagiu, Elena Mateescu, Nastasia Belc, Oana-Alexandra Oprea, Gina-Pușa Pîrvu

**Affiliations:** 1 National Research & Development Institute for Food Bioresources—IBA Bucharest, 020323 Bucharest, Romania; nastasia.belc@bioresurse.ro (N.B.); gina.constantinescu@bioresurse.ro (G.-P.P.); 2 National Meteorological Administration (METEO—Romania), 013686 Bucharest, Romania; elena.mateescu@meteoromania.ro (E.M.); oprea@meteoromania.ro (O.-A.O.)

**Keywords:** *Fusarium* head blight (FHB), deoxynivalenol (DON), wheat grading, soil, drought, heavy precipitation, heavy flood, dipole and omega atmospheric blocks, Vb cyclone, climate change

## Abstract

This article assesses the occurrence of *Fusarium*-damaged kernels (FDKs) in common wheat (*Triticum aestivum*) under the influence of environmental factors and extreme weather events in Romania (exceptionally high air temperatures and extreme pedological drought produced by a dipole block in summer 2015, and extreme precipitation and floods produced by an omega block in spring 2016). Wheat samples (*N* = 272) were analyzed for FDKs via visual estimation and manual weighing according to ISO 7970 and are statistically evaluated using SPSS. The dipole block in 2015 reduced the effects of environmental factors to non-significant correlations with FDKs, while the omega block in 2016 was non-significantly to very significantly correlated with FDKs in the northwestern and western regions. The occurrence of FDKs was favored for wheat cultivation in acidic soils and inhibited in alkaline soils. Wheat samples with FDKs ≥ 1% were sampled from crops grown in river meadows with high and very high risks of flooding. Knowing the contaminants’ geographical and spatial distributions under the influence of regular and extreme weather events is important for establishing measures to mitigate the effects of climate change and to ensure human and animal health.

## 1. Introduction

Common wheat (*Triticum aestivum*) is the second most cultivated cereal after maize in regions between 30 and 60° N (Europe; North America, in the United States of America and southern Canada; Asia, in the Russian Federation, Kazakhstan, eastern China, northern India, Pakistan, Afghanistan, and Iran) and 25 and 40° S (South America, in southern Brazil, Bolivia, Paraguay, and northeastern Argentina; southern South Africa; Australia) [1,2,3]. An analysis of the global distribution of wheat cultivation regions showed the existence of several climate types based on the Köppen–Geiger classification in 1901–2010, namely sub-tropical, Mediterranean, oceanic, and continental climates [2,4]. According to the Food and Agriculture Organization (FAO), the average production shares of wheat by region in 1961–2020 were as follows: Asia 38.3%, Europe 36.5%, Americas 19.1%, Oceania 3.2%, and Africa 2.9% [1]. In 2015–2016, the top 10 wheat producers were China, India, the Russian Federation, the United States of America, France, Canada, Ukraine, Germany, Pakistan, and Australia, with average yields between 132,955,000 tonnes and 23,008,537 tonnes [1]. Romania had an average wheat production rate of 8,196,776 tonnes, with 7,962,421 tonnes in 2015 and 8,431,131 tonnes in 2016 [1].

Romania is a country located in Southeastern Europe, at a latitude of 46° N and longitude of 25° E [5]. It has a humid temperate continental climate according to the Köppen–Geiger climate classification, which is influenced by the Carpathian Mountains climate, the Mediterranean climate in the southwest, the Atlantic air masses in the west and northwest, the Scandinavian–Baltic and Arctic air masses in the north and northeast, the continental air masses in the east and southeast, the Black Sea and the Danube River climate in the southeast and south, and the Arabian Peninsula air masses in the southeast [4,6,7,8,9]. The agricultural soils in Romania are Chernozem, Phaeozem, and Luvisol, which vary in their chemical and physical properties [10,11,12]. Chernozems are found in the plains and are very favorable for wheat cultivation [11,12]. Chernozems have pH levels ranging from neutral to basic due to the high contents of organic matter and CaCO_3_, high porosity, good moisture-holding capacity, and increased aridity due to the semi-arid to dry sub-humid climate; they are conducive to the contamination of cereals with fungi of the *Aspergillus* and *Penicillium* genera [10,13]. Phaeozems are found in Mureș and Cluj counties (Transylvania) and Olt county (Oltenia Plain) and are Chernozem-like soils, although with degraded characteristics [11,12]. Luvisols are found in the sub-Carpathian regions and are unfavorable for wheat cultivation [11,12,14]. Luvisols have a pH range of slightly acid to very acid due to the lower content of organic matter, lack of CaCO_3_, low porosity, low moisture-holding capacity, and low aridity due to the sub-humid and humid climate; they are conducive to contamination of cereals with fungi of the genus *Fusarium* [10,15]. These soils are some of the major soil types in Europe: Chernozem soils are found in the steppe area of Eastern and Southeastern Europe (southern parts of European Russia, Ukraine, the Republic of Moldova, and Romania, as well as in northern Serbia); Phaeozem soils are found in the very humid area of Central Europe (especially in Switzerland, Austria, Slovakia, and Hungary) and Luvisol soils are found in the sub-Alps, sub-Carpathian region, the sub-Balkan plateaus, and in the high hills [10,12]. 

**Figure 1:**
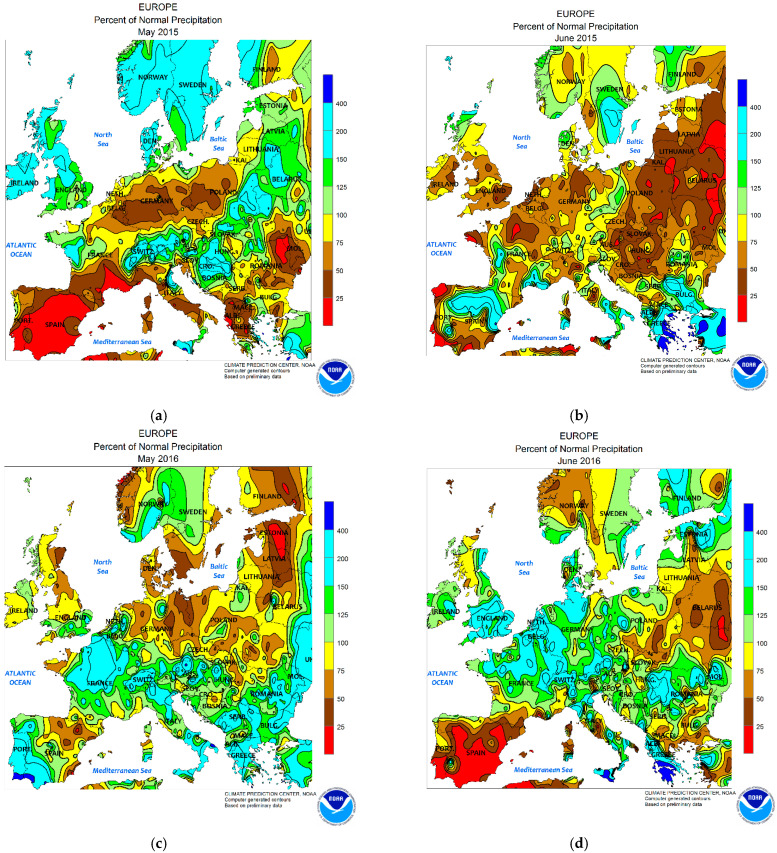
Spatial and geographic distributions of precipitation in Europe in 2015 and 2016, according to the National Oceanic and Atmospheric Administration (NOAA): (**a**,**b**) precipitation in May and June in 2015; (**c**,**d**) precipitation in May and June in 2016 [22]. Atmospheric blocking events: dipole block in May–August in 2015 [16] and omega block between 26 May and 8 June in 2016 [26,27,28].

In 2015–2016, Europe experienced different extreme weather events, namely exceptionally high air temperatures and extreme and severe levels of pedological drought in May–August in 2015 and extreme precipitation and floods in May–June in 2016 (Figure 1) [16,17,18,19,20,21,22]. A weather event is classified as extreme (heavy precipitation or floods, heat waves, drought, extratropical or tropical cyclones, cold waves, heavy snowfall, storm surges, tornadoes, ice, or hail storms) when it has extreme values of meteorological importance, such as rare rates of occurrence, e.g., a 100 year return value, magnitude, temporal duration, and timing, as well as spatial scale and multivariate dependencies, and causes loss of life and economic damages [23]. A brief description of these extreme weather events and their effects on agriculture is necessary because climate projections for Europe by 2100 suggest an increase in the frequency of extreme and heavy precipitation caused by rising sea surface temperatures, an increased risk of floods and storms, and an increased risk of drought during the summer [24,25]. 

In May–August in 2015, Europe recorded maximum air temperatures above 37 °C (London, 36.7 °C; Paris, 39.7 °C; Berlin, 37.9 °C; Kitzigen, in Germany, 40.3 °C; Catania, in Italy, 42.8 °C; Córdoba, in Spain, 45.2 °C; Dobřichovice, in the Czech Republic, 39.8 °C; Yerevan, in Armenia, 40.9 °C) and extreme pedological drought (Figure 1a,b) [16,17]. In Romania, the year 2015 was one of the extremely dry years in the 21st century when the south and west of the country recorded between 39 and 50 consecutive days with air temperatures above 32 °C between 1 June and 31 August; a maximum air temperature of 40.7 °C was recorded in Giurgiu on 30 July [29,30]. The summer of 2015 was characterized by a dipole-like structure (atmospheric blocking event) with a precipitation deficit and climatological drought in the central and southern parts of Europe, and with above-average precipitation in the northern and northwestern parts of Europe and Fennoscandia [16]. The most affected regions were the central and eastern parts of Europe and the northern Balkans (southern Germany, southwestern Poland, the Czech Republic, Slovakia, Austria, Romania, Ukraine, and European Russia) and impacts continued in some regions even into 2016; Belgium and the Netherlands also recorded meteorological droughts [16,31]. The 2015 European droughty summer was among the six hottest and driest summers since 1950, and it was more or less due to climate change; it was classified as an extreme weather event because it caused severe socioeconomic impacts in various water-related sectors in Europe [16,17,31].

From 26 May to 8 June 2016, Europe recorded extreme and heavy precipitation and floods in France, Belgium, the Netherlands, Germany, and Austria from 26 May to 5 June; in the United Kingdom from 7 to 8 June; and in Romania and the Republic of Moldova from 2 to 3 June (Figure 1c,d) [18,19,20,32,33,34]. The most affected regions were central France and southern Germany [18,19]. The weather events were associated with an omega block pattern (atmospheric blocking event) and were classified as extreme due to economic and life losses [16,17,35]. 

Atmospheric blocking is a large-scale disturbance that spreads very slowly and persists for a long time in the middle- and high-latitude flows. Based on the shape of the jet stream, there are three configurations for blocking flows: the omega block, the dipole block, or the rex block [35]. In the Northern Hemisphere, atmospheric blocking is favored over the Atlantic region during the negative or positive phase of the North Atlantic Oscillation (NAO); it forms in the region from about 30° W to 60° E and persists for about 8–10 days, with a frequency of occurrence of approximately 30–35 blocking events per year [35,36,37,38]. An omega (Ω) block is a synoptic pattern that occurs at medium to high latitudes, which extends in 1–3 days and obstructs the regular progress from west to east in migratory cyclones and anticyclones (Figure 1c,d) [22,26,27,28,36,39,40]. Because of their size, omega blocks are often quite persistent and can lead to flooding and drought conditions depending on one’s location under the patterns; cooler temperatures and precipitation accompany the low patterns, while warm and clear conditions prevail under the high patterns [39]. Blockage and stagnation of cyclones and anticyclones prevent the dispersion of air pollutants so that high values of pollution are recorded above the main hotspots in Europe (Italy in Po Valley, Poland in Silesia Province, northern France, Benelux, the south of the United Kingdom) [35,41]. The type of pollutant influences the chemistry of the precipitation, which has adverse effects on the environment (vegetation, water, and soil pH), thereby influencing the *Fusarium* spp. growth and deoxynivalenol production in cereals [41,42,43,44].

High humidity and wet weather accompanied by warm temperatures during the wheat anthesis favor spike infection with fungi of the *Fusarium* genus, development of *Fusarium* head blight (FHB) disease, and mycotoxin production (deoxynivalenol, zearalenone, fumonisins, nivalenol, moniliformin, T-2/HT-2 toxin, diacetoxyscirpenol) [45,46,47,48,49,50]. By improving the resistance to FHB disease, wheat can have five resistance components: type 1—resistance to initial infection; type 2—resistance to disease spread within a spike; type 3—resistance to kernel infection (assessed by counting the proportion of visibly damaged kernels, FDKs); type 4—tolerance to *Fusarium* spp.; type 5—resistance to toxins by decomposition or inactivation [50,51,52,53]. 

The *Fusarium*-damaged kernels (FDKs) are defined as grains whose pericarp is contaminated with *Fusarium* mycelium; these kernels often have a slightly faded appearance, are wrinkled, and have diffuse pink or white spots with an indefinite outline. Grains attacked by fusariosis are the main source of mycotoxins [54,55,56,57,58,59]. FDK measurements can be performed through visual estimation and manual counting or weighing, near-infrared spectroscopy (NIR), image-based optical sorting, and digital imaging seed phenotyping. Evaluations of these methods have shown that visual estimation and manual counting or weighing of FDKs have medium to strong and direct correlations with deoxynivalenol [53,60,61,62,63]. Contamination with fungi and mycotoxins in wheat FDKs can continue post-harvest, during transport or storage, and can be reduced by physical, chemical, or biological methods without being completely eliminated [64,65,66,67]. In the baking industry, wheat with 3% FDKs contains a high level of protease, which does not affect the volume of bread but deteriorates the properties of the gluten [68]. The consumption of food and animal feed made from wheat containing FDKs and mycotoxins promotes disease in humans and animals [46,47]. 

The global regions where *F. graminearum* and deoxynivalenol occur have a Köppen–Geiger climate type that corresponds to the major mild temperate group, with the oceanic, humid sub-tropical, and Mediterranean sub-types (Europe; North America, in eastern United States; southeastern South America, in southeastern Brazil and northeastern Argentina; southeastern South Africa and Australia; eastern China) [4,10,45,48,69,70,71,72,73,74]. The chemotypes and species of *Fusarium* spp. and deoxynivalenol occurrence in grains have a geographic distribution that is correlated with climatic conditions [10,73]. The National Aeronautics and Space Administration (NASA) has forecast a 17% increase in wheat crop yields by 2100 and an expansion of cultivation areas in the northern United States and Canada, the plains of northern China, Central Asia, southern Australia, and eastern Africa [75]. In Europe, raising the air temperature by 2 °C to 5 °C will increase the risk of wheat contamination, with aflatoxins produced by *Aspergillus* spp. in the west, south, and southeast (including southeastern Romania); conversely, the contamination with *Fusarium* spp. and deoxynivalenol will decrease proportionally in these regions, increasing in the northern and eastern regions [76]. In Romania, climate change forecasts indicated an increase in air temperature and a decrease in precipitation in the eastern Southern Plain, Oltenia Plain, and southern Moldavia, while changes in the Western Plain and sub-Carpathian regions will not be evident [77].

This article aims to assess the occurrence of *Fusarium*-damaged kernels (FDKs) in wheat under the influence of environmental factors in Romania in the years 2015 and 2016 with extreme weather events. FDK measurement were performed via visual estimation and manual weighing. Romania’s data were used for knowledge transfer and to facilitate an understanding of the occurrence of FDKs in wheat and other cereals in Europe. This paper is the first article reporting FDKs in wheat in two consecutive years with different extreme weather events (exceptionally high air temperatures and extreme pedological drought in 2015 in summer; extreme precipitation and floods in 2016 in spring) from a multidisciplinary approach (fungi, mycotoxin, climatology, agro-meteorology, agronomy, pedology, hydrology, and geography). The assessment of the FDKs in wheat in Romania in 2015 and 2016 with extreme weather events comes in support of the research on deoxynivalenol occurrence in triticale and other cereals (winter wheat, durum wheat, maize, rye, oats, and sorghum) in Romania and fourteen other European countries in the 2012–2014 period with extreme weather events [10]. These articles can help increase the scientific knowledge and raise awareness about the effects of extreme weather events, agroclimatic conditions, and environmental factors on the occurrence of *Fusarium* spp. and deoxynivalenol contamination in cereals, food, and animal feed. The data will be useful to scientific researchers, legislative regulators, commodities producers, and traders affected by climate change. 

## 2. Results

### 2.1. Agrometeorologic Factors in Romania, in the Agricultural Years 2015 and 2016

**Figure 2:**
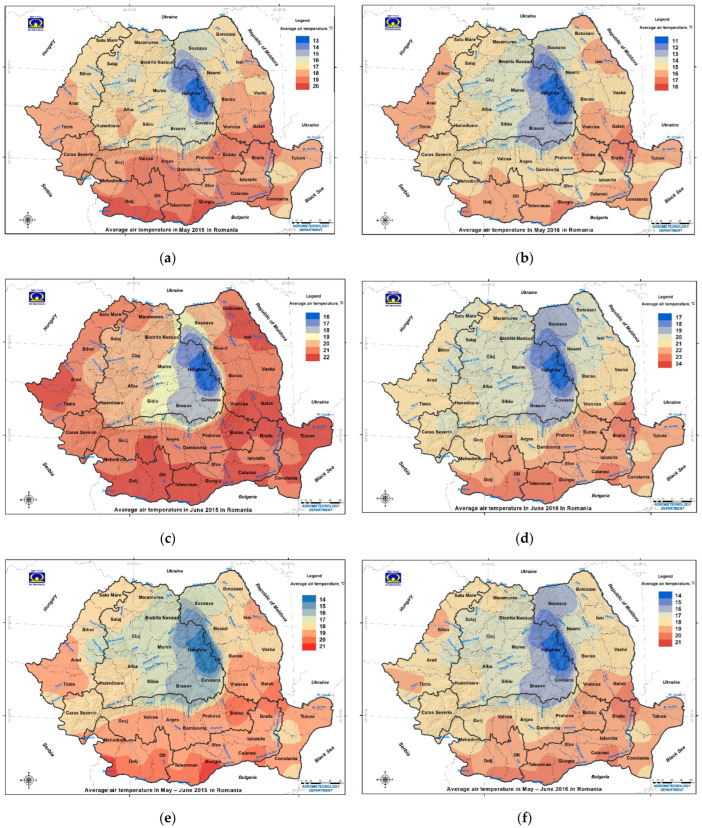
Spatial and geographic distributions of the air temperatures in Romania in 2015 and 2016: (**a**,**b**) average air temperature in May and June 2015; (**c**) average air temperature in May–June 2015; (**d**,**e**) average air temperature in May and June 2016; (**f**) average air temperature in May–June in 2016.

**Figure 3:**
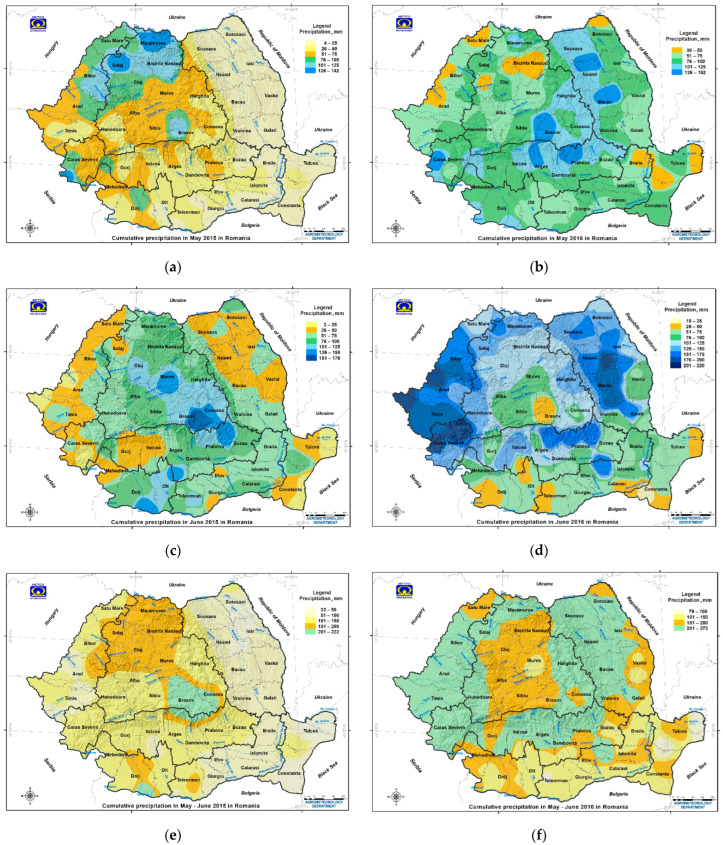
Spatial and geographic distributions of precipitation in Romania in the agricultural years 2015 and 2016: (**a**,**b**) cumulative precipitation in May and June 2015; (**c**) cumulative precipitation in May–June 2015; (**d**,**e**) cumulative precipitation in May and June 2016; (**f**) cumulative precipitation in May–June 2016.

The agricultural year 2015 was characterized by a mild autumn and a mild winter, followed by a typical spring (March–May: average air temperature of 16 °C and cumulative precipitation of 960 mm) and a hot summer (June–August: average air temperature of 22.5 °C and cumulative precipitation of 1691 mm) (Figure S1). During the critical period of wheat growth (May–June), an average air temperature of 18 °C, cumulative precipitation of 766 mm, and total water reserve in the soil of 839 m^3^/ha were recorded (Figure 2, Figure 3 and Figure S1). The characteristic of the agricultural year 2015 was the extreme or severe pedological drought, which manifested even in the regions with higher humidity (Transylvania, Southern Hilly Area, and Western Plain) (Figure 1a,b, Figure 2a–c, Figure 3a–c and Figure S1) [29,30]. 

The agricultural year 2016 was characterized by a mild autumn and a warm winter, followed by a rainy spring (March–May: average air temperature of 16.7 °C and cumulative precipitation of 1325 mm) and a hot summer (June–August: average air temperature of 22.1 °C, cumulative precipitation of 2309 mm). During the critical period of wheat growth (May–June), an average air temperature of 18.3 °C, cumulative precipitation of 1298 mm, and total water reserve in the soil of 1051 m^3^/ha were recorded (Figure 1c,d, Figure 2d–f, Figure 3d–f and Figure S1). In June, the highest cumulative precipitation values were recorded in the Western Plain, Moldavia, Southern Hilly Area, and Transylvania (Figure 1c,d and Figure 3d–f). In Romania, the omega block caused extreme and heavy precipitation in the Moldavia region on 2 and 3 June, so the National Meteorological Administration (NMA) issued an orange warning code for Suceava, Botoșani, Neamț, Iași, Bacău, Vaslui, and Vrancea counties, and a yellow warning code for Galați county [32]. For these reasons, the National Institute of Meteorology and Hydrology (NIMH) issued red and orange codes in hydrographic basins affected by extreme and heavy precipitation in Moldavia and yellow codes in hydrographic basins in eastern Transylvania and the eastern Southern Hilly Area [32,33]. The agricultural year 2016 was rainy, with extreme and heavy precipitation and floods being produced by the omega block that affected Europe between 26 May and 8 June (Figure 1c,d and Figure S1b) [18,19,20,32,33,34]. 

In the dry year 2015, the cumulative precipitation in May–June was very significantly correlated with the eastern longitude (*r_xy_* = −0.502 ***), the historical de Martonne aridity index (*r_xy_* = 0.589 ***), and the historical climatic water deficit (*r_xy_* = 0.616 ***) (Tables S8 and S9). The average air temperature in May–June was very significantly correlated with the historical de Martonne aridity index (*r_xy_* = −0.623 ***) and the historical climatic water deficit (*r_xy_* = −0.667 ***) (Tables S8 and S9). In the rainy 2016, the cumulative precipitation in May–June was correlated with the eastern longitude (*r_xy_* = −0.310 *) and the historical climatic water deficit (*r_xy_* = 0.368 *) (Tables S8 and S9). The average air temperature in May–June was significantly correlated with the northern latitude (*r_xy_* = −0.471 **), very significantly correlated with the historical de Martonne aridity index (*r_xy_* = −0.608 ***), and the historical climatic water deficit (*r_xy_* = −0.688 ***) (Tables S8 and S9). 

The typical cycle of common wheat growth by month and the sum of the temperature for the vegetation period in Romania are as follows: September—sowing, germination/emergence, 120–130 °C; October—sowing, germination and emergence, third leaf, 130–150 °C; November—sowing, germination and emergence, third leaf, twinning, 200–250 °C; December–February—vernalization; April—twinning, stem elongation, 400–500 °C; May—stem elongation, boot, heading and anthesis, 400–500 °C; June—boot, heading and anthesis, physiological maturity, 700–800 °C; July– physiological maturity, harvesting, 1900–2100 °C. The optimum time to sow common wheat is between 25 September and 5 October in the Southern Hilly Area, the northern part of the country, and the intra-Carpathian depressions, and between October 1 and 10 for the southern part of the country (Dobrogea, Southern Plain, and Oltenia Plain), the Western Plain, and the Transylvanian Plain (NMA data). 

### 2.2. Fusarium-Damaged Kernels in Common Wheat in Romania in 2015 and 2016

#### 2.2.1. FDKs in Common Wheat by Agricultural Year and Agricultural Region in Romania in 2015 and 2016

**Figure 4:**
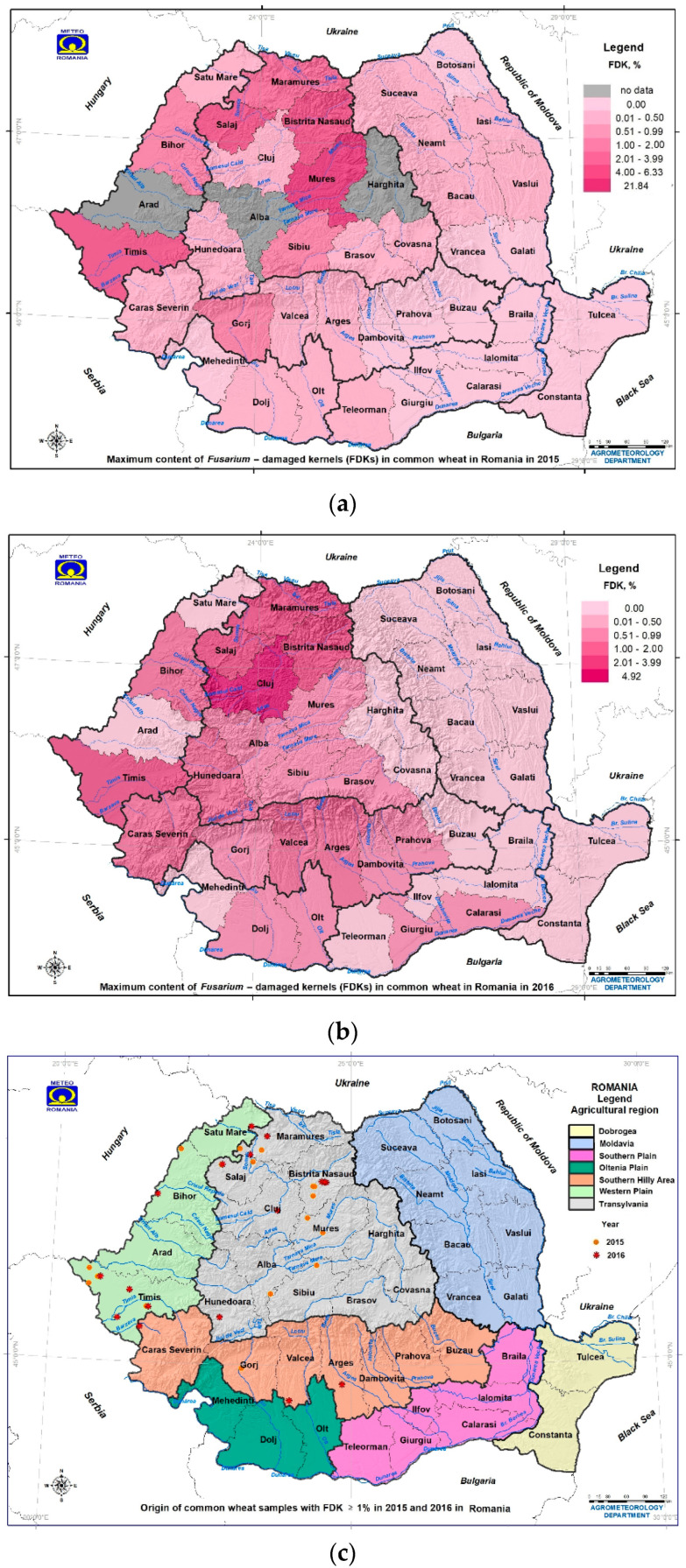
Spatial and geographic distributions of the maximum values of *Fusarium*-damaged kernels (FDKs) in common wheat in Romania in 2015 and 2016: (**a**,**b**) maximum values of FDKs in wheat by county and agricultural region in the dry part of 2015 and the rainy part of 2016; (**c**) origin of wheat samples with FDKs ≥ 1% in 2015 and 2016.

In the dry part of 2015, 135 wheat samples were analyzed, of which 56% were positive for FDKs and 13% were infected above the ML. The FDKs in wheat ranged within 0–21.84%, with an average of 0.45 ± 2.03% (Table 1 and Table S1). Values of FDKs in wheat above the ML were recorded in Transylvania with a maximum of 21.8% (Maramureș, maximum 2.36%; Sălaj, maximum 6.33%; Bistrița-Năsăud, maximum 4.05%; Mureș, maximum 21.8%; Sibiu, maximum 1.74%), in the Southern Hilly Area with a maximum of 1.37% (Gorj, maximum 1.37%), and in the Western Plain with a maximum of 5.63% (Timiș, maximum 5.63%; Bihor, maximum 1.79%) (Table 1, Tables S1 and S2; Figure 4a,c and Figure S2). 

In the rainy year of 2016, 137 wheat samples were analyzed, of which 87% were positive for FDKs and 12% were infected above the ML. The FDKs in wheat ranged within 0–4.92%, with an average of 0.82 ± 0.98% (Table 1 and Table S1). The FDK values in wheat above the ML recorded in Transylvania reached a maximum of 4.92% (Maramureș, maximum 2.65%; Sălaj, maximum 2.80%; Bistrița-Năsăud, maximum 2.06%; Cluj, maximum 4.92%; Hunedoara, maximum 1.43%), in the Southern Hilly Area reached a maximum of 1.70% (Argeș, maximum 1.56%; Vâlcea, maximum 1.70%; Caraș-Severin, maximum 1.53%), and in the Western Plain reached a maximum of 5.63% (Timiș, maximum 5.63%; Bihor, maximum 1.79%) (Table 1, Tables S1 and S2; Figure 4b,c and Figure S2). 

In the dry year 2015, FDKs in wheat were non-significantly correlated with the cumulative precipitation and the average air temperature in May–June (Table S8). In the rainy year 2016, FDKs in wheat were non-significantly correlated with the cumulative precipitation and the average air temperature in May–June (Table S9).

#### 2.2.2. FDKs in Common Wheat by Geographic Position in Romania in 2015 and 2016

In the dry part of 2015, values of FDKs in wheat above the ML were recorded at the geographic coordinates of 45° N, 21° E (Western Plain—in Timiș, maximum 5.63%) and 46–47° N, 23–24° E (Transylvania—in Sălaj, maximum 6.33%; Mureș, maximum 21.84%) (Table 1 and Tables S1–S4; Figure 4a,c and Figure S2a,b). 

In the rainy year 2016, values of FDKs in wheat above the ML were recorded at the geographic coordinates of 45° N, 21° E (Western Plain—in Timiș, maximum 2.04%), 46–47° N, 23° E (Transylvania—in Hunedoara, maximum 1.43%; Cluj, maximum 4.92%; Sălaj, maximum 2.80%) (Table 1 and Tables S1–S4; Figure 4b,c and Figure S2a,b). 

In the dry year 2015, FDKs in wheat were non-significantly correlated with the northern latitude and the eastern longitude (Table S8). In the rainy year 2016, FDKs in wheat were non-significantly correlated with northern latitude and very significantly correlated with the eastern longitude (*r_xy_* = −0.526 ***) (Table S9).

#### 2.2.3. FDKs in Common Wheat by Soil in Romania in 2015 and 2016

In Romania, extreme weather events in 2015–2016 led to the highest values of FDKs being observed in wheat grown in Phaeozem soil (21.84%), followed by Luvisol (6.33%) and Chernozem (5.63%) (Table 1, Table 2, Tables S1, S2 and S5).

In the dry year 2015, the highest values of FDKs in wheat were in crops grown in Phaeozem soil (Luvic–Phaeozem) (100% positive samples, 25% samples above the ML, maximum 21.84%), followed by Luvisol (60% positive samples, 13.33% samples above the ML, maximum 6.33%) and Chernozem (44.23% positive samples, 11.54% samples above the ML, maximum 5.63%). The high air temperatures and severe pedological drought in July–August 2015 determined an incidence rate of 56.30% positive samples and 13.33% samples above the ML (Table 1, Table 2, Tables S1, S2 and S5).

In the rainy year 2016, the highest values of FDKs in wheat were recorded in crops grown in Chernozem soil (78.26% positive samples, 10.87% samples above the ML, maximum 4.92%), followed by Luvisol (92.94% positive samples, 14.12% samples above the ML, maximum 2.80%) and Phaeozem (83.33% positive samples, 0% samples above the ML, maximum 0.56%). The extreme and heavy precipitation in May–June in 2016 determined an incidence rate of 87.59% positive samples and 12.41% samples above the ML of 1% (Table 1, Table 2, Tables S1, S2 and S5).

In Romania, the Chernozem soils are neutral and alkaline soils of the Eurasian Chernozem belt, the Phaeozem soils are acidic soils of Central Europe, and the Luvisol soils are acid and very acidic soils of the plateaus and high hills in Romania and Europe (Table 1, Table 2 and Table S1) [10,11,12]. 

#### 2.2.4. FDKs in Common Wheat by Aridity Indices in Romania in 2015 and 2016

##### De Martonne Aridity Index (Iar-dM) 

In the dry year 2015, values of FDKs in wheat above the ML were recorded in counties with a sub-humid climate (Western Plain—in Bihor, maximum 1.79%; Transylvania—in Mureș, maximum 21.84%) and counties with a humid climate (Transylvania—in Sălaj, maximum 6.33%; Bistrița-Năsăud, maximum 4.05%) (Table 1, Tables S1, S2 and S6; Figure 4a,c and Figure S2c,d).

In the rainy year 2016, values of FDKs in wheat above the ML were recorded in counties with a sub-humid climate (Western Plain—in Timiș, maximum 2.04%; Transylvania—in Cluj, maximum 4.92%) and the counties with a humid climate (Transylvania—in Sălaj, maximum 2.80%; Bistrița-Năsăud, maximum 2.06%) (Table 1, Tables S1, S2 and S6; Figure 4b,c and Figure S2c,d).

FDKs in wheat and historical Iar-dM values were non-significantly correlated in the dry year 2015 and significantly correlated in the rainy year 2016 (*r_xy_* = 0.367 **) (Tables S8 and S9). Historical Iar-dM values were non-significantly correlated with the northern latitude and significantly correlated with the eastern longitude (*r_xy_* = −0.420 **) (Tables S8 and S9).

##### Climatic Water Deficit (CWD) 

In the dry year 2015, values of FDKs in wheat above the ML were recorded in counties with a semi-arid climate (Western Plain—in Timiș, maximum 5.63%), a humid–balanced climate (Transylvania—in Mureș, maximum 21.84%), and humid climate (Transylvania–in Sălaj, maximum 6.33%) (Table 1, Tables S1, S2 and S7; Figure 4a,c and Figure S2c,d).

In the rainy year 2016, values of FDKs in wheat above the ML were recorded in counties with a semi-arid climate (Western Plain—in Timiș, maximum 2.04%), a humid–balanced climate (Transylvania—in Cluj, maximum 4.92%), and a humid climate (Transylvania—in Hunedoara, maximum 1.43%; Sălaj, maximum 6.33%; Southern Hilly Area—in Caraș-Severin, maximum 1.53%) (Table 1, Tables S1, S2 and S7; Figure 4a,c and Figure S2c,d).

In both years, the highest values of FDKs in wheat were determined in the regions with humid, humid–balanced, and sub-humid climates (Transylvania, Southern Hilly Area, and the Western Plain) (Table 1, Tables S1, S2 and S7; Figure 4 and Figure S2). In regions with a sub-humid climate (Oltenia Plain), semi-arid climate (Moldavia), and arid climate (Southern Plain and Dobrogea), the maximum values of FDKs in wheat varied between 0–0.22% in the dry year 2015 and 0–0.85% in the rainy year 2016 (Table 1 and Table S1; Figure 4 and Figure S2). 

FDKs in wheat and the historical CWD values were non-significantly correlated in the dry year 2015 and significantly correlated in the rainy year 2016 (*r_xy_* = 0.457 **) (Tables S8 and S9). Historical CWD was significantly correlated with the northern latitude (*r_xy_* = 0.421 **) and very significantly correlated with the eastern longitude (*r_xy_* = −0.555 ***) (Tables S8 and S9).

## 3. Discussion

### 3.1. Agrometeorologic Factors in Romania in the Agricultural Years 2015 and 2016

The agricultural years 2015 and 2016 with extreme weather events caused by atmospheric blocking (high air temperatures and extreme pedological drought in May–August in 2015 caused by a dipole block, and extreme precipitation and floods in May–June in 2016 caused by an omega block) were preceded by three agricultural years with other extreme weather events: 2012 with cold waves and heavy snowfall produced by a Siberian anticyclone in January–February, extreme precipitation and floods in May, and extreme air temperature and pedological drought in July–August; furthermore, 2013 and 2014 had extreme precipitation and floods produced by Vb cyclones in May–July [10,16,31,79,80,81,82,83,84,85,86,87,88,89,90]. The Vb cyclones and the dipole and omega blocks were favored by the North Atlantic Oscillations and correlated with the planetary wave resonance; they were more or less caused by climate change [6,8,17,79,80,89]. These atmospheric systems produced extreme weather events that influenced the aridity indices of the European regions and the occurrence of *Fusarium* spp. and deoxynivalenol in cereals [10]. 

In the critical period of May–June for grains in Romania, the average air temperature was non-significantly correlated with the FDKs in wheat in the dry year 2015 and the rainy year 2016, and very significantly correlated with the northern latitude in the rainy year 2016 and the historical aridity indices in both years (Figure 2; Tables S8 and S9). The average air temperature was significantly correlated with average deoxynivalenol in the triticale crops in 2012–2014 [10]. The cumulative precipitation in May–June was non-significantly correlated with FDKs in wheat in the dry year 2015 and the rainy year 2016, very significantly correlated with the eastern longitude and the historical aridity indices in 2015, inversely correlated with the eastern longitude, and non-significantly to moderate correlated with the historical aridity indices in in 2016 (Tables S8 and S9). Cumulative precipitation in May–June was distinctly and significantly correlated with the average deoxynivalenol in triticale crops in 2012–2014 (with historical precipitation and floods in May–July 2014; July was considered the fourth rainiest in the last 50 years in Romania) [10,85]. Maximum levels of deoxynivalenol contamination in wheat were 955 µg/kg in North Muntenia (Dâmbovița and Prahova counties) and 963.86 μg/kg in Brașov county in the dry year 2015, and 5483.94 µg/kg in Bistrița-Năsăud county in the rainy year 2016 [15,91,92]. In northern Poland, FHB incidence and disease severity were not correlated with air temperatures and only occasionally correlated with the cumulative precipitation in June–July in 2011–2013 [93]. These correlations show that average air temperature and cumulative precipitation in the critical period and high air temperature and extreme pedological drought in summer affect FDKs in wheat, depending on the extreme weather events. This hypothesis is supported by the occurrence of the FDKs in common wheat and deoxynivalenol mycotoxin in the cereal crops and animal feeds in Romania and other European countries in 2004–2018, with the level of contamination varying each year depending on the characteristics and environmental effects of the extreme weather events [10,48,69,70,71,73,74,91,92,94,95,96,97,98,99,100,101,102,103,104,105,106,107,108,109,110,111]. 

### 3.2. Fusarium-Damaged Kernels in Common Wheat in Romania in 2015 and 2016

#### 3.2.1. FDKs in Common Wheat by Agricultural Year and Region in Romania in 2015 and 2016

In the dry year 2015, the values of FDKs in wheat decreased (average 0.45 ± 2.03%) due to unfavorable dry weather conditions for crop infection in May–June (average air temperature of 18 °C and cumulative precipitation of 766 mm), determined by the dipole block (Figure 2a–c, Figure 3a–c and Figure 4a,c; Table 1, Tables S1 and S2). Wheat samples with FDKs below average were the most common, and those above the ML ranged within 1.37–21.84% and were recorded in the wetter counties of the Western Plain, Southern Hilly Area, and Transylvania (Table 1, Tables S1 and S2; Figure 4). The maximum values of deoxynivalenol contamination in wheat were registered in two neighboring counties, in the dry year 2015 (Brașov in southeastern Transylvania and Dâmbovița in the eastern Southern Hilly Area) and Bistrița-Năsăud in northwestern Transylvania in the rainy year 2016 [15,91,92]. The occurrence of FDKs in wheat in the dry year 2015 was demonstrated by non-significant correlations with agrometeorological factors in May–June (average air temperature and cumulative precipitation), in northern latitude with a significant correlation with historical aridity indices (Iar-dM and CWD), and a very significant correlation with eastern longitude (Figure 2, Figure 3 and Figure 4 and Figure S2; Table 1 and Tables S1–S8). The critical period May–June was followed by high air temperatures and extreme pedological drought in July–August, which did not favor the *Fusarium* infection and FDK and deoxynivalenol contamination in wheat (Table 1 and Table S1) [15,19,30,92]. In 2015, extreme pedological drought affected the whole of Europe, and the most affected regions were southern Germany, southwestern Poland, the Czech Republic, Slovakia, Austria, Romania, Ukraine, and European Russia; cereal crops were severely affected and had low productivity [16,17,21,29,30,31]. Therefore, cereals had low contamination levels with mycotoxins produced by *Fusarium* spp. (deoxynivalenol, zearalenone and fumonisin) and high contamination levels with mycotoxins produced by *Aspergillus* spp. and *Penicillium* spp. (aflatoxins and ochratoxin) [96,97,99,102,103,104]. In the previous year 2014, the occurrence levels of *Fusarium* spp., deoxynivalenol, zearalenone, and their derivatives in cereal crops and animal feed in Romania and many European countries were very high because of the extreme precipitation and floods produced by the stationing of Vb cyclones named Tamara and Yvette in May–July [10,69,94,95,96,97,98,99,100,101,102,103,104,105,106,107,108,109,110,111]. 

In the rainy year 2016, the values of FDKs in wheat were higher (average 0.82 ± 0.98%) due to the wetter weather conditions for crop infection in May–June (average air temperature of 18.3 °C and cumulative precipitation of 1298 mm), determined by the omega block (Figure 1, Figure 2d–f, Figure 3d–f, Figure 4b,c, and Figure S1; Table 1, Tables S1 and S2). The occurrence of FDKs in wheat in the rainy year 2016 was demonstrated by the very significant correlation with eastern longitude, significant correlations with historical aridity indices, and non-significant correlations with average air temperature in May–June and with northern latitude (Figure 2, Figure 3 and Figure 4 and Figure S2; Table 1 and Tables S1–S8). Values of FDKs in wheat above the ML ranged within 1.70–4.92% and were recorded in the regions with a wetter climate (Transylvania, Southern Hilly Area, and Western Plain, the most affected regions by the omega block in Romania) (Table 1, Tables S1 and S2; Figure 4 and Figure S2). In Poland, humid conditions in June 2016 delayed grain harvesting, influencing the level of *Fusarium* spp. and deoxynivalenol contamination [112]. 

The two-factor ANOVA without replication showed non-significant differences in the average FDKs in common wheat by geographic coordinates, agricultural region, and agricultural year in Romania in 2015–2016 (*p*-value > 0.05). The dry 2015 was marked by a higher incidence rate of the FDKs in wheat in the geographic area between 45.5–47° N and 23–24° E (the area is bounded by plateaus and high hills located between high mountain peaks, the “pole of the rain” and the “pole of the cold” in Romania, respectively), which also shows the effect of local precipitation on grain contamination in risk areas. In the rainy year 2016, this situation may also have been associated with the fact that the extreme and heavy precipitation and floods produced by the omega block on 2 to 3 June were of a lower magnitude and duration, with delayed timing and a lower spatial scale (in northern Romania) compared to the historical extreme precipitation and floods produced by the Vb cyclones in May–July 2014 throughout the country (Figure 1, Figure 2, Figure 3 and Figure 4, Figures S1 and S2; Table 1, Tables S1 and S2) [10,18,19,20,31,32,33,34,79]. It is emphasized that the Vb cyclones affected Central, Southeastern, and Eastern Europe in June in 2013 and May–July in 2014, high air temperature and extreme pedological drought affected the whole of Europe in July–August in 2012, the dipole block affected the whole of Europe and especially central and eastern parts and the northern Balkans in May–August in 2015, and the omega block affected Northwestern and Northern Europe in May–June in 2016 [10,16,17,21,22,30,31,33,36,79,80,81,82,83,84,85,86,87,88]. The effects of these extreme weather events on mycotoxin contamination in cereal crops were also reflected in the animal feed in Europe (mycotoxins produced by *Fusarium* spp. in the rainy years 2013, 2014, and 2016, and mycotoxins produced by *Aspergillus* spp. and *Penicillium* spp. in the dry years 2012 and 2015) [69,111,113,114]. 

The weather events recorded every year and the agro-climatic characteristics of the region influenced the crops indicators, including the production and yield, organoleptic and sanitary properties, physico-chemical indicators, impurities, and technological indicators. These indicators are important in the grading process of cereal crops, which is performed for commercial purposes. The FDKs in common wheat are included in the “impurities” category and the Romanian Grading Plan for common wheat for human consumption includes three grades: grade I—maximum 0.3%; grade II—maximum 0.5%; grade III—maximum 1% [54,55,56,57,58,59,78,115]. In the dry year 2015, the common wheat quality was very good in terms of FDKs: 81% of the samples were grade I; 6% of samples were grade II; 4% of samples were grade III; 10% of samples were not acceptable for human consumption (Figure S3). In the rainy year 2016, the common wheat quality was very good and good: 61% of the samples were grade I; 10% of samples were grade II; 17% of samples were grade III; 12% of samples were not acceptable for human consumption (Figure S3). Then, there was a 20% decrease in grade I of common wheat quality, a 5% increase in grade II, and a 13% increase in grade III, while unacceptable wheat for human consumption increased by only 2% and only in the regions with a humid climate. These small changes in common wheat quality gradation were due to the prolonged effects of the 2015 drought and short-lived heavy precipitation in late May and early June in 2016 (Figures S1–S3) [16,31,32,78,115]. Extrapolating the above points, the grading process of wheat quality in the European countries affected by the dry summer of 2015 and by the extreme precipitation and floods in the spring of 2016 was influenced by the geographic locations of the countries under the atmospheric blocking ridges [39]. Compared to the period 1961–2020, the average wheat production rates in the dry year 2015 and in the rainy year 2016 increased, especially in Asian countries with large agricultural areas (mainland China, India, and the Russian Federation), but also in North America (the United States of America, and Canada), Europe (France, Germany, and Ukraine), Pakistan, Australia, and Turkey. The average production share of wheat by global region in 2015–2016 was as follows: Asia 43.5%, Europe 34.2%, Americas 15.6%, Oceania 3.1%, and Africa 3.5% [1]. In Europe, the extreme dry summer 2015 reduced the wheat yields in the southeast, and short-lived extreme precipitation and floods in the 2016 spring reduced the wheat yields in Western and Northern Europe [1]. Romania and other Southeastern and Eastern European countries showed an increasing evolution of average wheat production in 2013–2016, and decreased production and yield in 2012 because of the extreme pedological drought recorded during the summer [1]. 

#### 3.2.2. FDKs in Common Wheat by Geographic Position in Romania in 2015 and 2016

In 2015 and 2016, the values of FDKs in common wheat above the ML were recorded in Transylvania, the Southern Hilly Area, and the Western Plain, which are located between 44.89–47.44° N and 21–26.11° E (Table 1 and Tables S1–S4; Figure 4 and Figure S2a,b). This spatial and geographic distribution of maximum values of FDKs in common wheat coincides with the geographic position of the highest values of deoxynivalenol contamination in common wheat in the dry year 2015 and the rainy year 2016 (Dâmbovița, Brașov, and Bistrița-Năsăud) [15,91,92]. FDKs in common wheat were correlated with the geographic position in both years, namely a non-significant correlation with northern latitude in the dry year 2015 and very significant correlation with eastern longitude in the rainy year 2016 (*r_xy_* = −0.526 ***) (Tables S8 and S9). Moreover, wheat samples with FDKs above the ML were sampled from localities on the banks of tributaries or affluents of the Danube River, where heavy precipitation and flooding were recorded in May–June in 2015 and 2016 (Figure 4c; Table S2). In 2016, which had short-lived heavy precipitation and floods in May–June, FDKs and deoxynivalenol in common wheat had similar geographic distributions, namely higher occurrence rates in Transylvania, the Southern Hilly Area, and the Western Plain (FDKs 1.70–4.92%; DON 1544–5483.94 µg/kg) and lower occurrence rates in the Oltenia Plain, Moldavia, Southern Plain, and Dobrogea (FDKs 0.15–0.52%; DON < 18.50–470.54 µg/kg) [91]. Extensive data on the occurrence of deoxynivalenol contamination in common wheat in Romania in the dry year 2015 and the rainy year 2016 will be published in a future article. In the 2012–2014 period, which had extreme weather events, Transylvania and the Southern Hilly Area regions represented a first-class division of average deoxynivalenol rates in triticale due to the significant correlation with average air temperature in May, with distinct and significant correlations with cumulative precipitation in May; the Western Plain, Oltenia Plain, Moldavia, the Southern Plain, and Dobrogea regions represented a second-class division of average DON values in triticale soils due to non-significant differences between deoxynivalenol levels [10]. 

The Western Plain and northern Transylvania are parts of the Pannonian Basin (which extends over several countries, including the whole of Hungary, eastern Austria, western Slovakia, southern Czech Republic, southeastern Poland, southwestern Ukraine, western Romania, northern Serbia, northeastern Slovenia, and northeastern Croatia), and their climatic characteristics are influenced by the Mediterranean, Atlantic, and Scandinavian–Baltic climates and the mountain climates. The Carpathian Mountains, the Alps and the Dinaric Alps represent a barrier to the circulation of these large-scale air masses, and the plateaus and high hills are areas conducive to *Fusarium* spp. and deoxynivalenol contamination in cereals (Figure 4 and Figure S2) [10,69,70,71,94,95,96,97,98,99,100,101,102,103,104,105,106,107,108,109,110,111].

In Romania, the areas with the highest occurrence rates of *Fusarium* spp. and deoxynivalenol contamination in cereals are located between 46–48° N and 23–24° E (Maramureș, Bistrița-Năsăud, Sălaj, and Mureș counties), close to the critical area in Europe between 49–51° N and 15–20° E (southern Germany, the Czech Republic, western Slovakia, and southeastern Poland) in 2004–2018 [10]. In the dry year 2015 and the rainy year 2016, the southern regions with a warm temperate continental climate and aridity ranging from sub-humid to semi-arid and arid climates (Oltenia Plain, Southern Plain, and Dobrogea), as well as the eastern region with a cold and semi-arid temperate continental climate (Moldavia), recorded sporadic occurrence of FDKs in wheat (Table 1 and Tables S1–S4; Figure 4 and Figure S2). In the dry year 2015, other European countries reported low levels of contamination with *Fusarium* spp. and deoxynivalenol in cereals due to the extreme drought in summer, including Bosnia and Herzegovina and Serbia in Southeastern Europe, the Czech Republic in Central Europe, and the Netherlands in Northwestern Europe [16,21,31,96,97,102,103,104,106]. In the rainy year 2016, Lithuania in Eastern Europe reported *Fusarium* spp. and deoxynivalenol contamination in spring cereals under the influence of precipitation in June and delayed harvesting [112]. The low level of deoxynivalenol contamination in cereals in the dry year 2015 was noted in the Netherlands and the Czech Republic, in spite of the extreme summer drought, although these countries recorded high and very high levels of contamination with *Fusarium* spp. and deoxynivalenol in wheat, maize, and animal feed in the extremely rainy years 2013 and 2014 [10,16,31,98,102,106,108]. The 2015 summer was the driest summer in North Slovakia and the second driest summer of the last 50 years (after 2003) in the Czech Republic and Poland; in the Netherlands and Belgium, a 1-in-20 year meteorological drought occurred from April to August [31]. In Poland, FHB was strongly correlated with disease severity and weakly correlated with FDKs in wheat; there was a strong correlation between disease severity and FDKs in 2011–2013 [93].

In Europe, the incidence and severity of the FHB disease and deoxynivalenol contamination in cereals decreased from north to south and from west to east, with decreasing air temperature and total precipitation increasing the agro-climatic aridity [10,73]. This spatial and geographical distributions are favored by the fact that the highest long-term total precipitation rates are produced by Atlantic and Mediterranean cyclones, while the lowest rates are produced by continental and polar cyclones [6,8,10,48,73,116]. Moreover, the epidemiological data for *Fusarium* species in Europe in 2000–2013 showed a geographic distribution of chemotypes, with variations between years and crops (wheat, maize, barley, oats, other *Poaceae*, soil): *F. graminearum* 15-ADON was dominant in Western, Central, and Southeastern Europe and rarely found in Northern Europe; *F. graminearum* 3-ADON was detected in Northern Europe, being dominant in Norway; *F.* *graminearum* and *F. culmorum* NIV-chemotype were detected in Western Europe and dropped rapidly further eastwards [73]. In Eastern Europe, FHB is expected in the Northern Caucasus; is sporadic in Southwestern Ukraine, the Republic of Belarus, the Baltic countries, central and northwestern Russia, and the Urals; and the disease is absent in Central Asia and Kazakhstan [117]. In recent years, FHB disease and deoxynivalenol, zearalenone, and T-2/HT-2 toxins have been observed in common wheat in Poland, Eastern Europe, West Siberia, and Northern Kazakhstan due to the global warming process [48,93,117,118,119].

Although Romania did not contribute epidemiological data to the European Database of *F. graminearum* and *F. culmorum* Trichothecene Genotypes [73], it was observed that the occurrence rates of *Fusarium* species and chemotypes in common wheat in 2005–2009 corresponded to those at the European level in 2000–2013: *F. graminearum* 15-ADON predominated (85%) and produced three times less DON than *F. culmorum* 3-ADON isolates; none of the isolates produced NIV [120,121,122]. Additionally, *F. graminearum* was dominant (75%) in stored wheat in Timiș county, even in the dry year 2011–2012 and the rainy year 2012–2013 [123]. This was due to Romania’s geographic location in Southern Europe and local and large-scale weather events. In Romania, the years 2011–2015 were part of a series of the sixteen warmest years in 1901–2015, of which 2013 and 2014 were among the extremely rainy years, and 2011–2012 and 2014–2015 were among the extremely dry years [124]. For Romania, by corroborating the data on the occurrence of FDKs in common wheat with the epidemiological data on *Fusarium* chemotypes in Romania and Europe and with the meteorological classification of 2011–2016 years, it can be assumed that *F. graminearum* was dominant in the very rainy 2013, 2014, and 2016 years and had lower incidence rates in the dry years 2012 and 2015. In future studies, it is intended to determine the dynamics of FDKs in grains in the years 2000–2016 and to identify the species of *Fusarium* in common wheat in the context of climate change.

#### 3.2.3. FDK Values in Common Wheat by Soil in Romania in 2015 and 2016

In the dry year 2015, the highest incidence of positive samples and the average and maximum values of FDKs in common wheat were detected in crops grown in Phaeozem soils (Luvic–Phaeozem) from Mureș county, followed by Luvisol soils and Chernozem soils (Table 1, Table 2, Tables S1, S2 and S5; Figure 2 and Figure 4a,c). The Luvic–Phaeozem in Mureș county presented the highest average deoxynivalenol contamination rates in triticale crops in the 2012–2014 period, with extreme weather events [10]. Analysis of the official catalogues of the Ministry of Agriculture and Rural Development containing quality data for grains crops in Romania showed that Mureș county recorded other very high values for FDKs in 2010 (maximum 28.13% in wheat), but lower attack rates in 2008 and 2011 (maximum 7.43% in wheat and 5.77% in triticale) [125,126,127]. These differences in FDKs in grains grown in Luvic–Phaeozem soils can be attributed to the chemical and physical properties of soils, agro-climatic conditions, large-scale weather events, and agricultural practices applied by grain growers. In 2012–2014, the occurrence rates of deoxynivalenol in triticale, common wheat, durum wheat, and rye crops in Romania in terms of the incidence and level of contamination were highest in crops grown in acidic soils such as Luvisol soils and Luvic–Phaeozem soils [10]. The highest levels of contamination with deoxynivalenol in wheat in the dry year 2015 and the rainy year 2016 were recorded in counties where Luvisol soils are dominant [15,91,92]. The low contamination levels reported by the Netherlands and the Czech Republic in 2015 were due to the extreme drought in summer and the locations of these countries within the trajectory and intersection of Atlantic and Mediterranean air masses, causing rich precipitation that increases soil acidification and promotes *Fusarium* spp. attack and deoxynivalenol production in cereals [7,12,31,42,43,44,102,106,128].

Although in the extremely rainy year of 2014, the countries in Southeastern Europe (Romania, in the Southern Plain, Dobrogea, and Oltenia Plain regions; Serbia in the Vojvodina region; Bosnia and Herzegovina; Croatia) reported massive levels of contamination with fusariotoxins in wheat and maize, they did not report high contamination levels in the dry year 2015 [10,96,97,99,103,104]. This fact shows the effects of alternating periods with extreme humidity and extreme drought (the changing water balance) on the soil microbiome [42,43,44]. In southern Romania and northeastern Serbia Chernozem soils are dominant, and their return to alkaline pH inhibited the fungus development cycle in the extremely dry year 2015. However, *Fusarium* spp. spores can survive for four years on cereal residues incorporated into the soil and can be activated by prolonged heavy precipitation after acidification of the soil [43,44].

In the rainy year 2016, the highest occurrence rates of FDKs in common wheat were recorded in crops grown in Luvisol soils (Table 1, Table 2, Tables S1, S2 and S5; Figure 2 and Figure 4b,c). The maximum value of FDKs in wheat was recorded in Cluj county, where Phaeozem soils predominate (Tables S1 and S2). The short-lived extreme or heavy precipitation produced by the omega block in Europe from 26 May to 8 June in 2016 affected the whole of Romania from 2 to 3 June, but Transylvania, Moldavia, the Southern Hilly Area, and the Western Plain regions recorded heavy precipitation rates of 100–373 mm (Figure 1c,d, Figure 3d–f and Figure S1). In these agricultural regions, both acidic soils (Luvisol soils and Phaeozem soils) and alkaline soils (Chernozem soils) can be found (Table 1, Tables S1 and S2). Due to the short duration of the heavy precipitation, the precipitation volume (maximum 218.5 mm in Timiș county), and spatial and geographic distributions of heavy precipitation and floods, the FDKs in wheat varied greatly in terms of the incidence rates of positive samples, but not of samples above the ML of 1% (Table 1, Tables S1 and S2; Figure 2d–f, Figure 3d–f and Figure 4b,c). Although the omega block affected several European countries that have both acid and alkaline soils (France, Germany, Belgium, Austria, and the Republic of Moldova), those countries have not published data on the occurrence rates of *Fusarium* spp. and deoxynivalenol contamination in cereals (Figure 1) [10]. 

#### 3.2.4. FDKs in Common Wheat by Aridity Indices in Romania in 2015 and 2016

##### De Martonne Aridity Index (Iar-dM)

In the dry year 2015, the FDKs in common wheat occurred as a result of local factors and the regular circulation of large-scale air masses in western and northwestern Romania (Timiș, Bihor, Sălaj, Mureș, Bistrița-Năsăud, and Maramureș counties), where the sub-humid to humid climate causes frequent contamination of cereals. In this area, the maximum FDKs in wheat ranged from 0.27 to 21.8% in the dry year 2015 (Table 1, Tables S1, S2 and S6; Figure 4a,c and Figure S2c,d). The high humidity in the western and northwestern area is determined by the circulation of large-scale Mediterranean, Atlantic, Scandinavian–Baltic, and Arctic air masses; by the heights of the Carpathian Mountains, with snowmelt until July; the hydrological basin of the Someș river, which presents high and very high risks of flooding; and the so-called ”precipitation pole in Romania” in Bihor county and “cold pole in Romania” in Covasna and Harghita counties [6,7,8,9,129,130]. The western part of the country (Timiș county), having a humid–balanced climate, showed maximum FDK values in common wheat of 1.25–5.63%, as this area receives strong hot and humid Mediterranean climate influences (Figure 1, Figure 2a–c, Figure 3a–c and Figure 4a,c). Heavy precipitation in May–June in 2015 was followed by high air temperatures and extreme drought in summer throughout the country, and the effect of humidity on the FDKs in common wheat was counteracted by the high air temperature and sub-humid to semi-arid climates in the south (Oltenia Plain, southern Moldavia, Southern Plain, and Dobrogea regions) (Table S2) [30]. Prolonged and extreme precipitation in May–July in 2014 decreased the aridity in southern regions and favored the occurrence of *Fusarium* spp. and deoxynivalenol contamination in common wheat, durum wheat, triticale, and rye crops [10,85,131]. In the dry year 2015, these agricultural regions returned to aridity indices close to the historical values (Figure S1c), which did not favor fungal attack during wheat anthesis. The situation appeared to be similar in other European countries [10,96,97,99,103,104]. 

In the rainy year 2016, the maximum values of FDKs in common wheat were lower than in the dry year 2015 (4.92% vs. 21.8%), although Romania was under the influence of an omega block from 2 to 3 June (Table 1, Tables S1 and S2; Figure 1c–d, Figure 2d–f, Figure 3d–f, Figure 4 and Figure S2). The northwestern part of the country (Transylvania, in Sălaj, Bistrița-Năsăud, and Maramureș counties), having a cold sub-humid to humid climate, showed maximum values of FDKs in common wheat of 1.03–2.80% (Figure 4 and Figure S2; Tables S1–S4 and S6). The western (Western Plain, in Timiș county) and southwestern (Southern Hilly Area, in Caraș-Severin county) parts of the country, having a humid–balanced climate, showed maximum values of FDKs in common wheat of 1.09–1.53% (Figure S2). Although the cumulative precipitation rates in the critical period of May–June were higher in the rainy year 2016 compared to the dry year 2015, FDKs in common wheat showed lower values but a similar spatial and geographic distribution in 2016 (Table 1, Tables S1 and S6; Figure 1, Figure 2, Figure 3 and Figure 4, Figures S1 and S2). Again, the regions with sub-humid to semi-arid and arid climates (Oltenia Plain, Moldavia, Southern Plain, and Dobrogea) showed very low occurrence rates of FDKs in wheat, which ranged within 0–0.58% (Figure 4b,c and Figure S2). 

The occurrence of FDKs in common wheat in agricultural regions with low historical aridity (high values of Iar-dM) was confirmed via Pearson correlation coefficient, with a non-significant correlation in the dry year 2015 and significant correlation in the rainy year 2016 (*r_xy_* = 0.367 **) (Tables S8 and S9). Additionally, the average deoxynivalenol contamination in the triticale crop in Romania in 2012–2014 was distinctly and significantly correlated with historical Iar-dM [10]. The maximum deoxynivalenol contamination in wheat in the dry year 2015 and the rainy year 2016 was determined by heavy precipitation in areas with low aridity (Dâmbovița, Brașov, and Bistrița-Năsăud) [15,91,92].

##### Climatic Water Deficit (CWD)

The climate water deficit is the difference between potential and actual evapotranspiration, representing the amount of water that plants would use if available. This depends on the structure and permeability of the soil, the amount of snow, the timing of springtime snow melt, weather events (precipitation, floods, and drought) and the distribution of vegetation [132,133,134]. In Romania, the geographic disposition and the height of the Carpathian peaks, the proximity to the Black Sea and Danube River, and the circulation of large-scale air masses caused spatial variation in the climatic water deficit in 1900–2000 [7,9,10,11,133]. Thus, the regions of Moldavia, the Southern Plain, and Dobrogea have a semi-arid to arid climate, because they receive Scandinavian–Baltic, Siberian, continental, Black Sea, and Danube River climatic influences with low levels of precipitation [7,9,11,133]. The regions of Oltenia Plain, the Western Plain, the Southern Hilly Area, and Transylvania have sub-humid, humid–balanced, and humid climates, respectively, because they receive Mediterranean and Atlantic climatic influences, which bring high levels of precipitation [7,9,11,133]. Statistical analysis showed that historic CWD is very significantly correlated with historical Iar-dM (*r_xy_* = 0.832 ***) and eastern longitude (*r_xy_* = −0.555 ***) and significantly correlated with northern latitude (*r_xy_* = 0.421 **) (Tables S8 and S9). As arid soils in Romania, Chernozem soils are found in areas with arid, semi-arid, and sub-humid climates, while Phaeozem soils and Luvisol soils are found in areas with humid–balanced to sub-humid and humid climates [11,12,133]. 

Prolonged and extreme precipitation rates in May–July 2014 reduced the CWD and changed the water balance and pH values of Chernozem soils, favoring a wide and high occurrence range of FDKs in terms of wheat and mycotoxin contamination in cereal crops both in Romania and other European countries [10,42,135,136,137,138]. Despite the high spore masses on cereal residues across the country in the extremely rainy year 2014, the FDKs in common wheat were limited to wetter regions, both in the dry year 2015 and the rainy year 2016 (Transylvania, Southern Hilly Area, and Western Plain) (Table 1 and Tables S1–S7; Figure 4 and Figure S2). Although the Moldavia region recorded extreme and heavy precipitation and floods produced by the omega block on 2 and 3 June in 2016, the occurrence rate of FDKs in common wheat was very low due to the short-lived heavy precipitation and high level of historical aridity (Figure 2, Figure 3 and Figure 4 and Figure S2; Table 1, Table 2, Tables S1, S2 and S7); in addition, it may have been the prolonged effect of the extreme drought in summer 2015 [16,31]. The occurrence of FDKs in common wheat in agricultural regions with low historical aridity (low values of CWD) was confirmed by the Pearson correlation coefficient values, with a non-significant correlation in the dry year 2015 and significant correlation in the rainy year 2016 (*r_xy_* = 0.457 **) (Tables S8 and S9). Additionally, the average deoxynivalenol contamination rate in triticale crops in Romania in 2012–2014 was distinctly and significantly correlated with historical CWD [10].

Analysis of FDKs in common wheat in the dry year 2015 and the rainy year 2016 via the spatial and geographic distributions of agro-meteorological factors, agricultural soils, historical aridity indices in 1900–2000, and statistical analysis showed the importance of local factors, large-scale weather events, and their interactions. These data support the multivariate tests of between-subject effects (comparisons of the average of deoxynivalenol rates in triticale crops in 2012–2014 with extreme weather events, air temperature, cumulative precipitation, and soil moisture reserves (i.e., the physical context in which the contamination occurred) as dependent variables, as well as the agricultural region and agricultural year as fixed factors), which showed at least a significant correlation (*p*-value < 0.05) [10]. 

## 4. Conclusions

Studies on the occurrence of *Fusarium*-damaged kernels (FDKs) in common wheat in Romania in 2015–2016 showed that the incidence rates and levels of these contaminants were determined by extreme weather events, which varied in type and meteorological importance each year (exceptionally high air temperatures and extreme pedological droughts produced by a dipole block in May–August in 2015, and extreme precipitation and floods produced by an omega block in May–June in 2016). These atmospheric blocking systems were determined by the North Atlantic Oscillation and associated with the planetary wave resonance, and were more or less correlated with climate change.

The effects of these extreme weather events on agriculture depended on their spatial and geographic distribution and agroclimatic characteristics of the regions. In Romania, the highest values of FDKs in common wheat in 2015–2016 were recorded in northwestern Transylvania, the Southern Hilly Area, and the Western Plain, which have a humid to sub-humid temperate continental climate, similar to Central Europe. The dipole block in 2015 reduced the effects of environmental factors to non-significant correlations with FDKs, while the omega block in 2016 was non-significantly to very significantly correlated with FDKs in the northwestern and western regions of Romania. In the case of FDKs in 2015–2016 in common wheat, samples contaminated above the MLs were sampled from cereals grown in river meadows with high and very high risks of flooding. The occurrence of FDKs was favored by cereal cultivation in acidic soils and inhibited in alkaline soils. There was low or no contamination in common wheat grown in Chernozem soils in the dry year 2015 and the rainy year 2016, although cereals grown in these soils were massively contaminated in 2014. Moreover, extreme weather events affected cereal crops in terms of production, yield, and grading parameters.

Knowing the contaminants’ geographic and spatial distribution under the influence of regular and extreme weather events in recent years is important because climate change will increase contamination with mycotoxins produced by *Aspergillus* spp. and *Penicillium* spp. in Southern and Southeastern Europe and with mycotoxins produced by *Fusarium* spp. in Eastern and Northern Europe. Other factors will be integrated into this multidisciplinary approach, including agricultural technologies, fertilizers and herbicides, neighboring vegetation, hydro-technical structures, and environmental pollution.

## 5. Materials and Methods

### 5.1. Sampling 

Common wheat was sampled (*N* = 272) in July–August in 2015 and 2016 from the localities where grains were contaminated with DON ≥1000 µg/kg (*N* = 109/2826 samples) in the 2012–2014 period with extreme weather events [10,139,140,141]. Sampling was performed on wheat batches according to SR EN ISO 24333:2010/AC:2011 “Cereals and cereal products. Sampling’’, immediately after wheat introduction into the warehouses. The activity was performed by qualified staff from the Ministry of Agriculture and Rural Development (MARD). The wheat samples weighing 1 kg each were accompanied by data on the economic operator, wheat variety, lot size, and dates of harvest in the field and sampling in the warehouse. The samples from the arid Moldavia region were among the first to be sampled and brought into the laboratory in July, although that region was the most affected by the omega block on 2 to 3 June 2016. This proved that common wheat was not significantly affected by short-lived heavy precipitation and *Fusarium* head blight in the region. The cereals were dried to a moisture content of 14% before being placed in storage spaces if necessary. 

For the prevention and treatment of plant diseases, growers apply insecticides and fungicides approved by the National Phytosanitary Authority, which is the specialized body of the Ministry of Agriculture and Rural Development in the field of plant protection, phytosanitary quarantine, and phytosanitary products [142]. Among the fungicides used for prevention and treatment of *Fusarium* head blight disease that are approved and widely used in Romania, we mention Nativo Pro 325 SC and Falcon^®^ Pro (Bayer SRL, Bucharest, Romania), Revicare^®^ (BASF SRL, Bucharest, Romania), and Kantik 450 EC (Syngenta Agro SRL, Bucharest, Romania).

### 5.2. Analysis of *Fusarium*-Damaged Kernels in Common Wheat 

The *Fusarium*-damaged kernels in common wheat were measured by visual observation and manual weighing and expressed as percentages according to SR EN ISO 7970:2009, because ISO 7970:2011 (the standard in-force during the study) did not contain the FDK parameter for wheat impurities [54]. The moisture content of the grains was not used in the calculation of the percentages of FDKs in common wheat because it is not requested by the standard. 

The maximum limit (ML) of FDKs in common wheat is 1% [78]. 

Analysis of FDKs in common wheat was performed by the National Research and Development Institute for Food Bioresources—IBA Bucharest. The evaluation method of the content of foreign bodies and seeds with defects (impurities) in consumer wheat was accredited by the Romanian Accreditation Association (RENAR), according to the standards ISO 7970, ISO 15587 and ISO 17025 in force during these years (Certificate no. LI 417 on 11 July 2011). The accreditation certificate in force since 19 November 2020, contains this analysis as well as the determination of the deoxynivalenol mycotoxin in cereals and feed by Ridascreen® DON test kit (R-Biopharm, Darmstadt, Germany) [10]. 

IBA Bucharest was designated as a reference laboratory for grain analysis following Government Decision no. 677 on 19 July 2001, which was repealed by Government Decision no. 546 on 9 June 2010. The institute holds the 2001–2011 grain quality catalogues (pdf), which will be used to analyse the dynamics of FDKs in grains in the context of climate change. 

### 5.3. Geographic Coordinates

The northern latitude and eastern longitude (degrees) of each Romanian county (*N* = 41) were determined using Google Earth [5], and were grouped by agricultural region (*N* = 7; Transylvania, Southern Hilly Area, Moldavia, Oltenia Plain, Western Plain, Southern Plain, and Dobrogea) based on agroclimatic factors. 

The agricultural regions are delimited in the maps of Romania in Figure 2, Figure 3 and Figure 4 and clearly named in Figure 4c. 

### 5.4. Agroclimatic Data

Agrometeorological factors (air temperature, °C; precipitation, mm; soil moisture reserve, m^3^/ha) were recorded by the official network of Meteorological Weather Stations (*N* = 159) from 1 September 2014 to 31 August 2016. Stations are equipped with Ceres-Wheat and Decision Support System for Agrotechnology Transfer, DSSAT v.3.5. software (University of Florida, Florida State, The United States of America). The network of automatic stations belonging to the National Meteorological Administration is connected to the International Meteorological Telecommunication System, ensuring the connectivity, operative transfer, and processing of primary data. 

The dominant agricultural soil types (Chernozem, Phaeozem, and Luvisol) in each county were set at a scale of 1:1,500,000 according to the Soil Atlas of Europe [12], which ensured statistical analysis at the regional level in Romania. Data on the soil fertilization, pH correctors, and other agricultural practices in Romania in 2015 and 2016 are not known. 

The aridity indices (de Martonne aridity index—Iar-dM, mm °C^−1^; climatic water deficit—CWD, mm H_2_O) of each county were estimated based on historical data in the 1900–2000 period [133,134,143] to determine the correlation with *Fusarium* spp. and deoxynivalenol on a long-term basis [10]. 

### 5.5. Data Processing and Statistical Analysis

All data were collected in an Excel file with the following variables: FDKs in common wheat sampes, years (2015 and 2016, with extreme weather events), agricultural region, county, geographic coordinates (northern latitude, eastern longitude), and agroclimatic data (agrometeorological factors—air temperature, precipitation, and soil moisture reserves; soil types—Chernozem, Phaeozem, and Luvisol; aridity indices—Iar-dM and CWD).

The influences of the geographic position and agroclimatic conditions on FDKs in common wheat were determined through statistical analysis using SPSS v.23 software (Statistical Package for the Social Sciences software with ANOVA and Pearson correlation) (IBM, Armonk, NY, USA). The probability was considered to be statistically significant at *p*-value ≤ 0.05.

### 5.6. Spatial and Geographic Distribution

The spatial and geographic distributions of air temperature, precipitation, and *Fusarium*-damaged kernels in common wheat in Romania in 2015 and 2016 were assessed using Geographic Information System (GIS) technology, using the Open Source ArcMap program version 10.1 (The Environmental Systems Research Institute—ESRI, Redlands, CA, USA) [144]. 

## Figures and Tables

**Table 1:** *Fusarium*-damaged kernels (FDKs) in common wheat by geographic coordinates, soil, historical aridity indices, and county in Romania in 2015 and 2016.

Agricultural Region	Geographic Coordinates		Aridity Indices, 1900–2000		Soil (% Counties)			*Fusarium*-Damaged Kernels (FDKs) in Common Wheat by Geographic Coordinates, Soil, Aridity Indices and County in Romania in 2015 and 2016							
								2015				2016			
								No. of Samples, Incidence			Interval of FDKs in Common Wheat, Mean ± SD (Median), %	No. of Samples, Incidence			Interval of FDKs in Common Wheat, Mean ± SD (Median), %
	Latitude, ° N	Longitude, ° E	Iar–dM, mm °C^−1^	CWD, mm H_2_O	Chernozem	Phaeozem	Luvisol	Total	≥0.01%	≥1%		Total	≥0.01%	≥1%	
Dobrogea	44.6	28.5	20	–375	100	0	0	2	0 0%	0 0%	0.00	4	2 50%	0 0%	0.00–0.15 0.06 ± 0.07 (0.04)
Southern Plain	44.3	26.6	26	–258	83.33	0	16.67	7	1 14%	0 0%	0.00–0.08 0.01 ± 0.03 (0.00)	14	10 71%	0 0%	0.00–0.85 0.20 ± 0.25 (0.12)
Moldavia	46.8	26.9	28	–194	12.20	0.00	7.32	14	5 36%	0 0%	0.00–0.22 0.11 ± 0.25 (0.00)	17	14 82%	0 0%	0.02–0.31 0.11 ± 0.09 (0.09)
Oltenia Plain	44.4	23.7	37	–167	33.33	23.33	33.33	9	6 67%	0 0%	0.00–0.21 0.10 ± 0.09 (0.11)	10	7 70%	0 0%	0.07–0.52 0.25 ± 0.18 (0.21)
Western Plain	46.5	22.1	33	–150	4.88	0.00	4.88	37	24 65%	7 19%	0.00–3.08 0.21 ± 0.58 (0.00)	20	19 95%	5 14%	0.02–2.04 0.62 ± 0.58 (0.43)
Southern Hilly Area	45.1	24.7	39	–93	2.44	0	14.63	34	13 38%	1 3%	0.00–1.37 0.11 ± 0.25 (0.00)	35	32 91%	3 9%	0.03–1.70 0.38 ± 0.47 (0.17)
Transylvania	46.4	24.3	46	–32	0	4.88	21.95	32	27 84%	10 31%	0.00–21.84 2.23 ± 5.40 (0.38)	37	35 95%	9 24%	0.04–4.92 0.87 ± 0.99 (0.66)
Romania	45.7	25.2	33	–181	39.02	7.32	53.66	135	76 56%	18 13%	0.00–21.84 0.45 ± 2.03 (0.00)	137	119 87%	17 12%	0.00–4.92 0.82 ± 0.98 (0.50)

The maximum limit (ML) of *Fusarium*-damaged kernels in common wheat is 1% [78].

**Table 2:** *Fusarium*-damaged kernels (FDKs) in common wheat by soil and agricultural year in Romania in 2015 and 2016.

Soil (Scale 1:1,500,000)	*Fusarium*–Damaged Kernels (FDKs) in Common Wheat by Soil and Agricultural Year in Romania in 2015 and 2016. FDKs (%): Interval, Average ± SD (Median); Sample Incidence (%): Positive Samples (≥0.01%); Samples with FDK ≥1%		
	2015	2016	2015–2016
Chernozem	0.00–5.63 0.40 ± 1.01 (0.00) 23/52 (44.23%) 6/52 (11.54%)	0.00–4.92 0.38 ± 0.81 (0.09) 36/46 (78.26%) 5/46 (10.87%)	0.00–5.63 0.39 ± 0.92 (0.06) 59/98 (60.20%) 11/98 (11.22%)
Phaeozem (Luvic–Phaeozem)	0.02–21.84 3.19 ± 7.56 (0.46) 8/8 (100%) 2/8 (25%)	0.00–0.56 0.21 ± 0.19 (0.15) 5/6 (83.33%) 0/6 (0%)	0.00–21.84 1.91 ± 5.76 (0.23) 13/14 (92.86%) 2/14 (14.29%)
Luvisol	0.00–6.33 0.41 ± 0.97 (0.05) 45/75 (60%) 10/75 (13.33%)	0.00–2.80 0.50 ± 0.59 (0.27) 79/85 (92.94%) 12/85 (14.12%)	0.00–6.33 0.46 ± 0.79 (0.18) 124/160 (77.50%) 22/160 (13.75%)
Romania	0.00–21.84 0.57 ± 2.08 (0.05) 76/135 (56.30%) 18/135 (13.33%)	0.00–4.92 0.45 ± 0.66 (0.18) 120/137 (87.59%) 17/137 (12.41%)	0.00–21.84 0.51 ± 1.54 (0.13) 196/272 (72.06%) 35/272 (12.87%)

The maximum limit of *Fusarium*-damaged kernels in common wheat is 1% [78].

## Data Availability

Not applicable.

## Supplementary Materials

The following are available online at https://www.mdpi.com/article/10.3390/toxins14050326/s1: Figure S1: Agrometeorological parameters in agricultural regions in Romania in 2015 and 2016; Figure S2: Spatial and geographic distributions of maximum values of *Fusarium*-damaged kernels (FDKs) in wheat in Romania in 2015 and 2016; Figure S3: Wheat grading for *Fusarium*-damaged kernels (FDKs) in Romania in the dry year 2015 and the rainy year 2016, respectively; Table S1: *Fusarium*-damaged kernels (FDKs) in wheat by geographic coordinates, soil type, historical aridity indices, and county in Romania in 2015 and 2016; Table S2: Maximum levels of *Fusarium*-damaged kernels (FDKs) in wheat by geographic position (agricultural region, geographic coordinates, county, and locality), aridity indices (de Martonne, Iar-dM; climatic water deficit, CWD), hydrographic basin, and wheat variety in Romania in 2015 and 2016; Table S3: *Fusarium*-damaged kernels (FDKs) in wheat by geographic coordinates (northern latitude) in Romania in 2015 and 2016; Table S4: *Fusarium*-damaged kernels (FDKs) in wheat by geographic coordinates (eastern longitude) in Romania in 2015 and 2016; Table S5: *Fusarium*-damaged kernels (FDKs) in wheat by soil in Romania in 2015 and 2016; Table S6: *Fusarium*-damaged kernels (FDKs) in wheat by aridity— de Martonne aridity index values in Romania in 2015 and 2016; Table S7: *Fusarium*-damaged kernels (FDKs) in wheat by aridity—climatic water deficit values in Romania in 2015 and 2016; Table S8: Correlation between *Fusarium*-damaged kernels (FDKs) in wheat and the agrometeorological factors, historical aridity indices, and geographic coordinates in Romania in the dry year 2015 (Pearson correlation coefficient); Table S9: Correlation between *Fusarium*-damaged kernels (FDKs) in wheat and the agrometeorological factors, aridity indices, and geographic coordinates in Romania in the rainy year 2016 (Pearson correlation coefficient).

## References

[1] FAO FAOSTAT. https://www.fao.org/faostat/en/#data/QCL/visualize.

[2] Khadka K., Earl H.J., Raizada M.N., Navabi A. (2020). A Physio-Morphological Trait-Based Approach for Breeding Drought Tolerant Wheat. Front. Plant Sci..

[3] Lantican M.A., Braun M.J., Payne T.S., Singh T.P., Sonder K., Baum M., van Ginkel M., Erenstein O. (2016). Impacts of International Wheat Improvement Research 1994–2014.

[4] Chen D., Chen H.W. (2013). Using the Köppen classification to quantify climate variation and change: An example for 1901–2010. Environ. Dev..

[5] Google Earth Google Landsat. https://earth.google.com/web/.

[6] Hofstätter M., Blöschl G. (2019). Vb Cyclones Synchronized with the Arctic-/North Atlantic Oscillation. J. Geophys. Res. Atmos..

[7] Mîndrescu M., Grădinaru I. (2017). Central and Eastern Europe paleoscience symposium: From local to global. Past Glob. Chang. Mag..

[8] Hofstätter M., Chimani B., Lexer A., Bloschl G. (2016). A new classification scheme of European cyclone tracks with relevance to precipitation. Water Resour. Res..

[9] Apostol L. (2008). The Mediterranean cyclones—The role in ensuring water resources and their potential of climatic risk, in the east of Romania. Present Environ. Sustain. Dev..

[10] Gagiu V., Mateescu E., Dobre A.A., Smeu I., Cucu M.E., Oprea O.A., Alexandru D., Iorga E., Belc N. (2021). Deoxynivalenol occurrence in triticale crops in Romania during the 2012–2014 period with extreme weather events. Toxins.

[11] Stănilă A.-L., Dumitru M. (2016). Soils Zones in Romania and Pedogenetic Processes. Agric. Agric. Sci. Procedia.

[12] European Commission (EC) (2005). Soil Atlas of Europe, European Soil Bureau Network.

[13] Tabuc C., Taranu I., Calin L. (2011). Survey of moulds and mycotoxin contamination of cereals in South-Eastern Romania in 2008–2010. Arch. Zootech..

[14] Ion V. *Phytotechnics* (University Course, Book). Faculty of Horticulture. University of Agricultural Sciences and Veterinary Medicine in Bucharest, Romania, 2010; p. 143. https://www.horticultura-bucuresti.ro/images/pdf/Fitotehnie.pdf.

[15] Stanciu O., Juan C., Berrada H., Miere D., Loghin F., Mañes J. (2019). Study on Trichothecene and Zearalenone Presence in Romanian Wheat Relative to Weather Conditions. Toxins.

[16] Ionita M., Tallaksen L.M., Kingston D.G., Stagge J.H., Laaha G., Van Lanen H.A.J., Scholz P., Chelcea S.M., Haslinger K. (2017). The European 2015 drought from a climatological perspective. Hydrol. Earth Syst. Sci..

[17] Dong B., Sutton R., Shaffrey L., Wilcox L. (2016). The 2015 European heatwave. Bull. Am. Meteorol. Soc..

[18] World Weather Attribution Initiative (WWAI) (2016). Rainstorms in France and Germany, May 2016. 9 June 2016. Partners: Environmental Change Institute, University of Oxford; Royal Netherlands Meteorological Institute (KNMI); Red Cross Red Crescent Climate Centre. https://www.worldweatherattribution.org/european-rainstorms-may-2016/?fbclid=IwAR2v5GKsK7Tkd0Rsk7xKKGSIp47ajEYE4LSxzugZlQC8L-d7aXCzRqufrVg.

[19] Davies R. France—Thousands Evacuated after River Levels Hit 100 Year High. *FloodList*, 2 June 2016. https://floodlist.com/europe/france-rivers-levels-hit-100-year-high-june-2016?fbclid=IwAR1NY2cVy2p4LEvMW1OcUyUhNvAt_ZjbuyauJE8m7sB1YKGaXQ0k_zpb1d4.

[20] Alfieri L., Feyen L., Salamon P., Thielen J., Bianchi A., Dottori F., Burek P. (2016). Modelling the socio-economic impact of river floods in Europe. Nat. Hazards Earth Syst. Sci..

[21] World Meteorological Organization (WMO) (2015). 2015 Second Hottest Year on Record for Europe. https://public.wmo.int/en/media/news/2015-second-hottest-year-record-europe.

[22] National Oceanic and Atmospheric Administration (NOAA) Europe. Percent of Normal Precipitation in May and June in 2015 and 2016.

[23] Stephenson D.B., Diaz H.F., Murnane R.J. (2008). Definition, diagnosis, and origin of extreme weather and climate events. Climate Extremes and Society.

[24] European Academies Science Advisory Council (EASAC) Extreme Weather Events in Europe. Preparing for Climate Change Adaptation: An Update on EASAC’s 2013 Study. Statement. *Extreme Weather Events* 2018; pp. 1–8. https://easac.eu/media-room/press-releases/details/new-data-confirm-increased-frequency-of-extreme-weather-events-european-national-science-academies-urge-further-action-on-climate-change-adaptation/.

[25] European Academies Science Advisory Council (EASAC) Trends in Extreme Weather Events in Europe: Implications for National and European Union Adaptation Strategies. EASAC Policy Report 22 November 2013; ISBN 978-3-8047-3239-1. https://www.leopoldina.org/uploads/tx_leopublication/2013_Easac_Report_Extreme_Weather_Events.pdf.

[26] Wolf G., Czaja A., Brayshaw D.J., Klingaman N.P. (2020). Connection between sea surface anomalies and atmospheric quasi-stationary waves. J. Clim..

[27] Rutkin A. What’s Causing the Devastating Floods in France and Germany? 3 June 2016. https://www.newscientist.com/article/2092131-whats-causing-the-devastating-floods-in-france-and-germany/.

[28] The Guardian Omega Block is Nature’s Secret Weapon. 16 June 2016. https://www.theguardian.com/science/2016/jun/16/omega-block-natures-secret-weapon-weatherwatch.

[29] Oprea O.-A., Mateescu E., Barbu A., Tudor R. (2018). Extreme dry years in the 21st century at the level of the agricultural areas of Muntenia, Romania. Sciendo.

[30] National Meteorological Administration (NMA) (2015). Annual Report. https://www.meteoromania.ro/wp-content/uploads/raport/Raport-2015.pdf.

[31] van Lanen H.A.J., Laaha G., Kingston D.G., Gauster T., Ionita M., Vidal J.P., Vlnas R., Tallaksen L.M., Stahl K., Hannaford J. (2016). Hydrology needed to manage droughts: The 2015 European case. Hydrol. Process..

[32] Covaci I.E., Dragomir M. Inundațiile din Moldova de la începutul lunii iunie 2016 [Floods in Moldavia in Early June 2016]. *Revista Stiintifica a Administratiei Nationale de Meteorologie* 2016; pp. 14–26. https://www.meteoromania.ro/wp-content/uploads/revista_stiintifica/revistastiintifica2016.pdf.

[33] National Meteorological Administration (NMA) (2016). Annual Report. https://www.meteoromania.ro/wp-content/uploads/raport/Raport-2016.pdf.

[34] Mediafax (2016). Cod Rosu de Inundatii, Extins in noua Judete din Moldova si Prelungit pana Sambata dupa Amiaza (Harta-Video) [Red Flood Code, Extended to Nine Counties in Moldavia and Extended until Saturday Afternoon (Video-Map)]. https://www.mediafax.ro/social/cod-rosu-de-inundatii-in-moldova-doi-oameni-si-au-pierdut-viata-iar-peste-doua-sute-au-fost-evacuati-din-cauza-inundatiilor-harta-video-15421251.

[35] Lupo A.R. (2020). Atmospheric blocking events: A review. Ann. N. Y. Acad. Sci..

[36] Woollings T., Barriopedro D., Methven J., Son S.-W., Martius O., Harvey B., Sillmann J., Lupo A.R., Seneviratne S. (2018). Blocking and its Response to Climate Change. Curr. Clim. Chang. Rep..

[37] Meteorological Office (MetOffice) (2017). Blocking Weather Patterns (Video). Youtube. https://www.youtube.com/watch?v=Fr2EmBYDK_8.

[38] Shabbar A., Huang J., Higuchi K. (2001). The relationship between the wintertime North Atlantic Oscillation and blocking episodes in the North Atlantic. Int. J. Climatol..

[39] National Oceanic and Atmospheric Administration (NOAA) Basic Wave Patterns. National Weather Services. https://www.weather.gov/jetstream/basic.

[40] American Meteorological Society (AMS) (2012). Blocking. Gloss. Meteorol..

[41] Vautard R., Colette A., van Meijgaard E., Meleux F., van Oldenborgh G.J., Otto F., Tobin I., Yiou P. (2016). Attribution of wintertime anticyclonic stagnation contributing to air pollution in western Europe. Bull. Am. Meteorol. Soc..

[42] Slessarev E.W., Lin Y., Bingham N.L., Johnson J., Dai Y., Schimel J.P., Chadwick O.A. (2016). Water balance creates a threshold in soil pH at the global scale. Nature.

[43] Leplat J., Friberg H., Abid M., Steinberg C. (2012). Survival of *Fusarium graminearum*, the causal agent of Fusarium head blight. A review. Agron. Sustain. Dev..

[44] Young I., Ritz K. (2000). Tillage, habitat space and function of soil microbes. Soil Tillage Res..

[45] Shah L., Ali A., Yahya M., Zhu Y., Wang S., Si H., Rahman H., Ma C. (2018). Integrated control of Fusarium Head Blight and deoxynivalenol mycotoxin in wheat. Plant Pathol..

[46] Jimenez-Garcia S.N., Garcia-Mier L., Garcia-Trejo J.F., Ramirez-Gomez X.S., Guevara-Gonzalez R.G.C., Feregrino-Perez A.A. (2018). *Fusarium* mycotoxins and metabolites that modulate their production. Fusarium-Plant Dis. Pathog. Divers. Genet. Divers. Resist. Mol. Markers.

[47] Waalwijk C., Vanheule A., Audenaert K., Zhang H., Warris S., van de Geest H., van der Lee T. (2017). *Fusarium* in the age of genomics. Trop. Plant Pathol..

[48] Backhouse D. (2014). Global distribution of *Fusarium graminearum*, *F. asiaticum* and *F. boothii* from wheat in relation to climate. Eur. J. Plant Pathol..

[49] Trail F. (2009). For blighted waves of grain: *Fusarium graminearum* in the postgenomics era. Plant Physiol..

[50] Champeil A., Doré T., Fourbet J.-F. (2004). Fusarium head blight: Epidemiological origin of the effects of cultural practices on head blight attacks and the production of mycotoxins by *Fusarium* in wheat grains. Plant Sci..

[51] Mesterhazy A. (2020). Updating the Breeding Philosophy of Wheat to Fusarium Head Blight (FHB): Resistance Components, QTL Identification, and Phenotyping—A Review. Plants.

[52] Mesterhazy A. (1995). Types and components of resistance to Fusarium head blight of wheat. Plant Breed..

[53] Khaeim H.M., Clark A., Pearson T., Van Sanford D. (2019). Methods of assessing *Fusarium* damage to wheat kernels. Al-Qadisiyah J. Agric. Sci..

[54] Wheat (Triticum aestivum L.)—Specification.

[55] Wheat (Triticum aestivum L.)—Specification.

[56] Wheat (Triticum aestivum L.)—Specification.

[57] Cereal and Cereal Products—Determination of Besatz in Wheat (*Triticum aestivum* L.), Durum Wheat (*Triticum durum* Desf.), Rye (*Secale cereale* L.), Triticale (*Triticosecale Wittmack* spp.) and Feed Barley (*Hordeum vulgare* L.).

[58] Cereale și Produse Cerealiere. Determinarea Conținutului de Impurități în Grâu (*Triticum aestivum* L.), Grâu Durum (*Triticum durum* Desf.), Secară (*Secale cereale* L.), Triticale (*Triticosecale Wittmack* spp.) și orz Pentru Hrana Animalelor (*Hordeum vulgare* L.).

[59] Canadian Grain Commission Wheat: Grading Factors. https://www.grainscanada.gc.ca/en/grain-quality/official-grain-grading-guide/04-wheat/grading-factors.html.

[60] Ackerman A.J., Holmes R., Gaskins E., Jordan K.E., Hicks D.S., Fitzgerald J., Griffey C.A., Mason R.E., Harrison S.A., Murphy J.P. (2022). Evaluation of Methods for Measuring *Fusarium*-Damaged Kernels of Wheat. Agronomy.

[61] Alisaac E., Behmann J., Rathgeb A., Karlovsky P., Dehne H.-W., Mahlein A.-K. (2019). Assessment of *Fusarium* Infection and Mycotoxin Contamination of Wheat Kernels and Flour Using Hyperspectral Imaging. Toxins.

[62] Paul R.A., Lipps P.E., Madden L.V. (2005). Relationship between visual estimates of Fusarium Head Blight intensity and deoxynivalenol accumulation in harvested wheat grain: A meta-analysis. Phytopathology.

[63] Ochodzki P., Twardawska A., Wiśniewska H., Góral T. (2021). Resistance to Fusarium Head Blight, Kernel Damage, and Concentrations of *Fusarium* Mycotoxins in the Grain of Winter Wheat Lines. Agronomy.

[64] Edwards S.G., Kharbikar L.L., Dickin E.T., MacDonald S., Scudamore K.A. (2018). Impact of pre-harvest rainfall on the distribution of Fusarium mycotoxins in wheat mill fractions. Food Control.

[65] Belluco B., de Camargo A.C., da Gloria E.M., dos Santos Dias C.T., Button D.C., Calori-Domingues M.A. (2017). Deoxynivalenol in wheat milling fractions: A critical evaluation regarding ongoing and new legislation limits. J. Cereal Sci..

[66] Paterson R.R.M., Lima N. (2010). How will climat change affect mycotoxins in food?. Food Res. Int..

[67] Tirado M.C., Clarke R., Jaykus L.A., McQuatters-Gollop A., Frank J.M. (2010). Climate change an food safety: A review. Food Res. Int..

[68] Bacala R., Fu B.X., Cordova K., Hatcher D.W. (2021). Wheat *Fusarium* Protease Specificity and Effect on Dough Properties. Foods.

[69] Gruber-Dorninger C., Jenkins T., Schatzmayr G. (2019). Global Mycotoxin Occurrence in Feed: A Ten-Year Survey. Toxins.

[70] Gagiu V., Mateescu E., Armeanu I., Dobre A.A., Smeu I., Cucu M.E., Oprea O.A., Iorga E., Belc N. (2018). Post-Harvest Contamination with Mycotoxins in the Context of the Geographic and Agroclimatic Conditions in Romania. Toxins.

[71] Gagiu V. (2018). Triticale Crop and Contamination with Mycotoxins under the Influence of Climate Change—Global Study. J. Hyg. Eng. Des..

[72] Trabucco A., Zomer R. (2019). Global Aridity Index and Potential Evapotranspiration (ET0) Climate Database v2. figshare. Fileset. CGIAR Consort. Spat. Inf. (CGIAR-CSI).

[73] Pasquali M., Beyer M., Logrieco A., Audenaert K., Balmas V., Basler R., Boutigny A.-L., Chrpová J., Czembor E., Gagkaeva T. (2016). A European Database of *Fusarium graminearum* and *F. culmorum* Trichothecene Genotypes. Front. Microbiol..

[74] Schatzmayr G., Streit E. (2013). Global occurrence of mycotoxins in the food and feed chain: Facts and figures. World Mycotoxin J..

[75] National Aeronautics and Space Administration (NASA) Impact of Climate Change on Global Wheat Yields. Visualizations by Mark SubbaRao, Released on 1 September 2021. https://svs.gsfc.nasa.gov/cgi-bin/details.cgi?aid=4914.

[76] Battilani P., Toscano P., van der Fels-Klerx H.J., Moretti A., Camardo Leggieri M., Brera C., Rortais A., Goumperis T., Robinson T. (2016). Aflatoxin B1 contamination in maize in Europe increases due to climate change. Sci. Rep..

[77] Busuioc A., Caian M., Bojariu R., Boroneant C., Baciu M., Dumitrescu A. (2012). Scenarii de schimbare a regimului climatic in Romania pentru perioada 2001–2030 [*Scenarios for changing the climate regime in Romania for the period 2001–2030*]. Brochure on the website of the Ministry of Environment, Waters and Forests. Admin. Naț. Met. București.

[78] Ministerul Agriculturii și Dezvoltării Rurale (MADR) (2017). MANUAL din 5 iulie 2017 de Gradare Pentru Semințe de Consum [MANUAL of 5 July 2017 Grading for Consumer Seeds]. Publicat în Monitorul Oficial nr. 537 bis din 10 iulie. http://legislatie.just.ro/Public/DetaliiDocumentAfis/192063.

[79] Stadtherr L., Coumou D., Petoukhov V., Petri S., Rahmstorf S. (2016). Record Balkan floods of 2014 linked to planetary wave resonance. Sci. Adv..

[80] Ionita M., Dima M., Lohmann G., Scholz P., Rimbu N. (2015). Predicting the June 2013 European Flooding Based on Precipitation, Soil Moisture, and Sea Level Pressure. J. Hydrometeorol..

[81] Schröter K., Kunz M., Elmer F., Mühr B., Merz B. (2015). What made the June 2013 flood in Germany an exceptional event? A hydro-meteorological evaluation. Hydrol. Earth Syst. Sci..

[82] Zurich Insurance Company (2015). Balkan Floods of May 2014: Challenges Facing Flood Resilience in a Former War Zone. A Zurich Flood Resilience Program Case Study. https://www.preventionweb.net/files/44332_44332zurichrisknexusmay2015balkanfl.pdf.

[83] International Commission for the Protection of the Danube River (ICPDR) (2014). Floods in June 2013 in the Danube River Basin. A Brief Overview of Key Events and Lessons Learned.

[84] Strelec Mahović N., Renko T., Tutiš V., Trošić T. (2015). Synoptic Analysis of the Catastrophic Floods in SE Europe, May 2014. Newsletter of the Working Group on Co-operation Between European Forecasters (WGCEF) N° 20. Eur. Forecast..

[85] Polifronie E.M. (2014). Luna iulie 2014—A patra cea mai ploioasă din ultimii 50 de ani [July 2014—The fourth Rainiest in the last 50 Years]. Rev. Științifică Admin. Naț. Meteorol. Bucuresti.

[86] World Meteorological Organization (WMO) (2013). Assessment of the Observed Extreme Conditions during Late Boreal Winter 2011/2012. World Meteorological Organization, Weather—Climate—Water, WCDMP.

[87] Bloschl G., Nester T., Komma J., Parajka J., Perdigão R.A.P. (2013). The June 2013 flood in the Upper Danube Basin, and comparisons with the 2002, 1954 and 1899 floods. Hydrol. Earth Syst. Sci..

[88] National Administration Romanian Waters (NARW) Raport ‘’Evaluarea Preliminară a Riscului la Inundații’’ 2010-2016 (Report ‘’Preliminary Flood Risk Assessment‘’ 2010–2016). Administrația Bazinală De Apă Buzău—Ialomița. http://www.inhga.ro/documents/10184/386602/PFRA_Report_RO5.pdf/1f0fae43-c468-4343-8d0e-695492c07368.

[89] Kautz L.-A., Martius O., Pfahl S., Pinto J.G., Ramos A.M., Sousa P.M., Woollings T. (2021). Atmospheric Blocking and Weather Extremes over the Euro-Atlantic Sector—A Review. Weather Clim. Dyn..

[90] Wu B., Yang K., Francis J.A. (2017). A Cold Event in Asia during January–February 2012 and Its Possible Association with Arctic Sea Ice Loss. J. Clim..

[91] Gagiu V., Mateescu E., Cucu M.E., Dobre A.A., Oprea O.A., Smeu I., Pîrvu G.P., Vătuiu I. (2017). Grain contamination with *Fusarium* sp. and deoxynivalenol under influence of the agro-climatic conditions from Romania in the year 2015–2016. Proceedings of the Communication Summaries of The Food Integrity Conference.

[92] Gagiu V., Doja L., Mateescu E., Smeu I., Cucu M.E., Dobre A.A., Oprea O., Iorga E., Belc N. (2016). Contamination with Deoxynivalenol in the Milling—Bakery Industry under the Influence of Climatic Conditions from Romania. J. Hyg. Eng. Des..

[93] Lenc L., Czecholiński G., Wyczling D., Turów T., Kaźmierczak A. (2015). Fusarium head blight (FHB) and *Fusarium* spp. on grain of spring wheat cultivars grown in Poland. J. Plant Prot. Res..

[94] Topi D., Babič J., Pavšič Vrtač K., Tavčar-Kalcher G., Jakovac-Strajn B. (2020). Incidence of *Fusarium* Mycotoxins in Wheat and Maize from Albania. Molecules.

[95] Leggieri M.C., Lanubile A., Dall’Asta C., Pietri A., Battilani P. (2020). The impact of seasonal weather variation on mycotoxins: Maize crop in 2014 in northern Italy as a case study. World Mycotoxin J..

[96] Kos J., Hajnal E.J., Malachová A., Steiner D., Stranska M., Krska R., Poschmaier B., Sulyok M. (2020). Mycotoxins in maize harvested in the Republic of Serbia in the period 2012–2015. Part 1: Regulated mycotoxins and its derivatives. Food Chem..

[97] Kos J., Hajnal E.J., Šarić B., Jovanov P., Nedeljković N., Milovanović I., Krulj J. (2017). The influence of climate conditions on the occurrence of deoxynivalenol in maize harvested in Serbia during 2013–2015. Food Control.

[98] Svoboda M., Blahová J., Honzlová A., Kalinová J., Macharáčková P., Rosmus J., Mejzlík V., Kúkol P., Vlasáková V., Mikulková K. (2019). Multiannual occurrence of mycotoxins in feed ingredients and complete feeds for pigs in the Czech Republic. Acta Vet. Brno.

[99] Udovicki B., Audenaert K., De Saeger S., Rajkovic A. (2018). Overview on the Mycotoxins Incidence in Serbia in the Period 2004–2016. Toxins.

[100] Bryła M., Waśkiewicz A., Podolska G., Szymczyk K., Jęedrzejczak R., Damaziak K., Sułek A. (2016). Occurrence of 26 Mycotoxins in the Grain of Cereals Cultivated in Poland. Toxins.

[101] Tima H., Berkics A., Hannig Z., Ittzés A., Nagy E.K., Mohácsi-Farkas C., Kiskó G. (2017). Deoxynivalenol in wheat, maize, wheat flour and pasta: Surveys in Hungary in 2008–2015. Food Addit. Contam. Part B Surveill..

[102] Van Der Fels-Klerx H., Adamse P., Punt A., Van Asselt E.D. (2018). Data Analyses and Modelling for Risk Based Monitoring of Mycotoxins in Animal Feed. Toxins.

[103] Pleadin J., Vasilj V., Petrovic D., Frece J., Vahcic N., Jahic S., Markov K. (2017). Annual variations of *Fusarium* mycotoxins in unprocessed maize, wheat and barley from Bosnia and Herzegovina. Croat. J. Food Sci. Technol..

[104] Pleadin J., Frece J., Lešić T., Zadravec M., Vahčić N., Staver M.M., Markov K. (2017). Deoxynivalenol and zearalenone in unprocessed cereals and soybean from different cultivation regions in Croatia. Food Addit. Contam. Part B Surveill..

[105] Vogelgsang S., Musa T., Bänziger I., Kägi A., Bucheli T.D., Wettstein F.E., Pasquali M., Forrer H.-R. (2017). *Fusarium* Mycotoxins in Swiss Wheat: A Survey of Growers’ Samples between 2007 and 2014 Shows Strong Year and Minor Geographic Effects. Toxins.

[106] Sumíková T., Chrpová J., Džuman Z., Salava J., Štěrbová L., Palicová J., Slavíková P., Stránská-Zachariášová M., Hajslova J. (2017). Mycotoxins content and its association with changing patterns of *Fusarium* pathogens in wheat in the Czech Republic. World Mycotoxin J..

[107] Aureli G., Amoriello T., Belocchi A., D’Egidio M., Fornara M., Melloni S., Quaranta F. (2015). Preliminary survey on the co-occurrence of DON and T2+HT2 toxins in durum wheat in Italy. Cereal Res. Commun..

[108] Chrpová J., Šíp V., Sumíková T., Salava J., Palicová J., Štočková L., Džuman Z., Hajslova J. (2016). Occurrence of *Fusarium* species and mycotoxins in wheat grain collected in the Czech Republic. World Mycotoxin J..

[109] Šliková S., Gavurnikova S., Hašana R., Minarikova M., Gregova E. (2016). Deoxynivalenol in Grains of Oats and Wheat Produced in Slovakia. J. Agric. For..

[110] Mankeviciene A., Jablonskytė-Raščė D., Maiksteniene S. (2014). Occurrence of mycotoxins in spelt and common wheat grain and their products. Food Addit. Contam. Part A.

[111] Kovasky P. (2015). Mycotoxin Survey 2014—Focus on Russian Poultry and Swine Feed. BIOMIN. https://www2.biomin.net/in-te/blog-posts/mycotoxin-survey-2014-focus-on-russian-poultry-and-swine-feed/.

[112] Kochiieru Y., Mankevičienė A., Ceseviciene J., Semaškienė R., Dabkevičius Z., Janavičienė S. (2020). The influence of harvesting time and meteorological conditions on the occurrence of Fusarium species and mycotoxin contamination of spring cereals. J. Sci. Food Agric..

[113] Rapid Alert System for Food and Feed (RASFF) RASFF Annual Reports. Reports and Publications. European Commission. https://ec.europa.eu/food/safety/rasff/reports_publications_en.

[114] National Sanitary Veterinary and Food Safety Authority (NSVFSA) Reports on the Implementation of the Single Multiannual National Plan for Integrated Control for Romania (2007–2019). http://www.ansvsa.ro/informatii-pentru-public/planul-national-multianual-unic-de-control-integrat-pentru-romania/#.

[115] Tamba-Berehoiu R.M., Popa C.N., Popescu S., Suciu A. (2012). The effect of the *Fusarium* sp. attack on the quality parameters of Romanian wheat. Sci. Bull. Ser. F Biotechnol..

[116] Messmer M., Gómez-Navarro J.J., Raible C. (2015). Climatology of Vb cyclones, physical mechanisms and their impact on extreme precipitation over Central Europe. Earth Syst. Dyn..

[117] AgroAtlas Project (2003). Project “Interactive Agricultural Ecological Atlas of Russia and Neighboring Countries. Economic Plants and their Diseases”. http://www.agroatlas.ru/en/content/diseases/Tritici/Tritici_Fusarium_graminiarum/index.html.

[118] Gagkaeva T., Gavrilova O., Orina A., Lebedin Y., Shanin I., Petukhov P., Eremin S. (2019). Analysis of Toxigenic *Fusarium* Species Associated with Wheat Grain from Three Regions of Russia: Volga, Ural, and West Siberia. Toxins.

[119] Nugmanov A., Beishova I., Kokanov S., Lozowicka B., Kaczynski P., Konecki R., Snarska K., Wołejko E., Sarsembayeva N., Abdigaliyeva T. (2018). Systems to reduce mycotoxin contamination of cereals in the agricultural region of Poland and Kazakhstan. Crop Prot..

[120] Cornea C.P., Israel-Roming F., Ciuca M., Voaides C. (2013). Natural occurrence of *Fusarium* species and corresponding chemotypes in wheat scab complex from Romania. Rom. Biotechnol. Lett..

[121] Ittu M., Cană L., Ciucă M., Voaideș C., Cornea P. (2012). Phenotypic and marker assisted evaluation of aggressiveness versus wheat in some Romanian *Fusarium* populations. Rom. Agric. Res..

[122] Ittu M., Cană L., Voica M., Lupu C. (2010). Multi-environment evaluation od disease occurence, aggressiveness and wheat resistance in wheat/*Fusarium* pathosystem. Rom. Agric. Res..

[123] Bozac P., Botău D., Popescu S., Boldura O., Alexa E. (2014). Distribution of *Fusarium* species in Timis County (Western Romania) in relation with environmental conditions. J. Hortic. For. Biotechnol..

[124] Mateescu E. The Romanian agrometeorological services and products—Current status and challenges in the context of climate change. Proceedings of the Workshop Agrometeorologists for Farmers in Hotter, Drier, Wetter Future.

[125] Ministerul Agriculturii, Padurilor si Dezvoltarii Rurale (2011). Calitatea Granelor din Recolta 2011, Nr. 16 (The Quality of the Grains in the Harvest 2011, No. 16).

[126] Ministerul Agriculturii, Padurilor si Dezvoltarii Rurale (2010). Calitatea Granelor din Recolta 2010, Nr. 15 (The Quality of the Grains in the harvest 2010, No. 15).

[127] Ministerul Agriculturii, Padurilor si Dezvoltarii Rurale (2008). Calitatea granelor din recolta 2008, Nr. 13 (The quality of the grains in the harvest 2008, No. 13).

[128] Mulder C., Van Wijnen H.J., Van Wezel A.P. (2005). Numerical abundance and biodiversity of below-ground taxocenes along pH gradient across the Netherlands. J. Biogeogr..

[129] European Spatial Planning Observation Network (ESPON) (2019). Flood Recurrence in Europe, Based on the Frequency of Floods in the Time Span of 1987–2002. ESPON Data Base. https://www.preventionweb.net/files/3827_Floodhazard8702N3.jpg.

[130] Micu D. (2009). Snow pack in the Romanian Carpathian under changing climatic conditions. Meteorol. Atmos. Phys..

[131] National Meteorological Administration (NMA) (2014). Annual Report. https://www.meteoromania.ro/wp-content/uploads/raport/Raport-2014.pdf.

[132] Flint A.E., Flint A.L., Thorne J.H. (2014). Climate Change: Evaluating Your Local and Regional Water Resources. U.S. Geol. Surv. (USGS) Fact Sheet.

[133] Paltineanu C., Mihailescu I.F., Seceleanu I., Dragota C., Vasenciuc F. (2007). Using aridity indices to describe some climate and soil features in Eastern Europe: A Romanian case study. Theor. Appl. Climatol..

[134] Allen R.G., Pereira L.S., Raes D., Smith M. (1998). Crop evapotranspiration. Guidelines for Computing Crop Water Requirements. FAO Irrigation and Drainage Paper 56.

[135] Furtak K., Grządziel J., Gałązka A., Gawryjołek K., Niedźwiecki J. (2021). Fungal biodiversity and metabolic potential of selected fluvisols from the Vistula River valley in Lubelskie, Poland. Appl. Soil Ecol..

[136] Siebielec S., Siebielec G., Klimkowicz-Pawlas A., Gałązka A., Grządziel J., Stuczyński T. (2020). Impact of Water Stress on Microbial Community and Activity in Sandy and Loamy Soils. Agronomy.

[137] Argiroff W.A., Zak D., Lanser C.M., Wiley M.J. (2016). Microbial Community Functional Potential and Composition Are Shaped by Hydrologic Connectivity in Riverine Floodplain Soils. Microb. Ecol..

[138] Ponnamperuma F.N. (1984). Effects of flooding on soils. Flooding and Plant Growth.

[139] Gagiu V., Mateescu E., Belc N. (2013). Risk Assessment of Cereal Crops Contamination with Deoxynivalenol in Romania, in 2012.

[140] Gagiu V., Cucu M., Dobre A., Mateescu E., Oprea O., Belc N. (2013). Contamination of the Wheat Crop with Deoxynivalenol Mycotoxin in the 2012–2013 Agricultural Year.

[141] Gagiu V., Cucu M., Dobre A., Mateescu E., Oprea O., Belc N. (2014). Contamination of Grain Crops with Deoxynivalenol in Romania, in Agricultural Year 2013–2014.

[142] National Phytosanitary Authority Regulatory Service for the Plant Protection—Pestexpert. https://www.anfdf.ro/.

[143] De Martonne E. (1926). Une nouvelle fonction climatologique: L’ indice d’aridité. Meteorologie.

[144] Environmental Systems Research Institute (ESRI) ArcGIS. Discover Your Power with ArcGIS. Redlands, CA, USA. https://www.esri.com/en-us/arcgis/about-arcgis/overview.

