# Supplementary material for: Assessment of Fusarium-Damaged Kernels in Common Wheat in Romania in the Years 2015 and 2016 with Extreme Weather Events
Source: Toxins (Basel). 2022 May 4;14(5):326. doi: 10.3390/toxins14050326 (PMC9145446; doi:10.3390/toxins14050326)
Supplement: Supplementary file 1 [file toxins-14-00326-s001.zip › toxins-1671646-supplementary.pdf]

toxins

MDPI

Supplementary Materials: Assessment of *Fusarium*-Damaged Kernels in Common Wheat in Romania in the Years 2015 and 2016 with Extreme Weather Events

Valeria Gagiu, Elena Mateescu, Nastasia Belc, Oana-Alexandra Oprea and Gina-Pușa Pîrvu

Note: An agricultural year is defined to start on 01 September and end on 31 August of the subsequent calendar year.

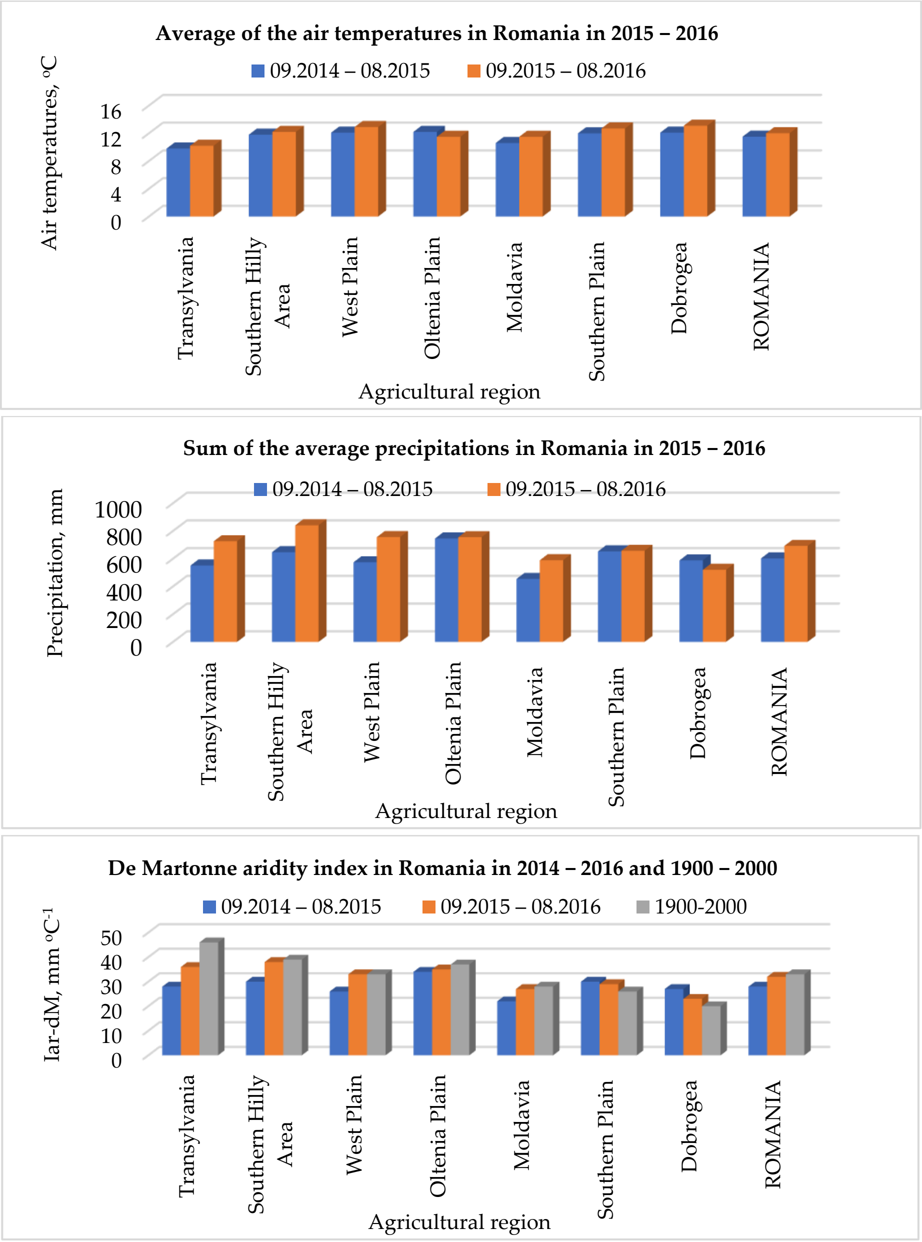

a) b) c)

Figure S1. Agrometeorological factors in agricultural regions in Romania in 2015 and 2016. Average of the air temperatures (**a**), Sum of the average precipitations (**b**), de Martonne aridity index, I_{ar}-dM (**c**).

toxins

MDPI

Table S1. *Fusarium*–damaged kernels (FDKs) in common wheat by geographic coordinates, soil type, historical aridity indices and county in Romania in 2015 and 2016.

County, Agricultural Region	Geographic Coordinates, degrees		Aridity Indices, in 1900–2000		Soil	*Fusarium*–Damaged Kernels (FDKs) in Common Wheat by Geographic Coordinates, Soil, Historical Aridity Indices and County in Romania in 2015 and 2016							
						2015				2016			
	Latitude, °N	Longitude, °E	Iar-dM, mm $^0C^{-1}$	CWD, mm H_2O		No. of Samples, Incidence			Interval of FDKs in Common Wheat, Mean ± SD (Median), %	No. of Samples, Incidence			Interval of FDKs in Common Wheat, Mean ± SD (Median), %
						Total	≥0.01%	≥1%		Total	≥0.01%	≥1%	
Maramureș	47.44	24.00	50	0	Lu	4	4	2	0.00 – 2.36<br>1.11 ± 1.26 (1.04)	4	4	3	0.69 – 2.65<br>1.57 ± 0.81 (1.47)
Bistrița-Năsăud	47.14	24.50	60	0	Lu	5	5	3	0.48 – 4.05<br>1.47 ± 1.48 (1.14)	7	7	3	0.20 – 2.06<br>1.04 ± 0.63 (0.89)
Sălaj	47.12	23.30	50	0	Lu	5	3	2	0.00 – 6.33<br>1.61 ± 2.74 (0.33)	5	5	1	0.12 – 2.80<br>0.85 ± 1.11 (0.49)
Mureș	46.59	24.61	40	–100	Ph	6	6	2	0.02 – 21.8<br>4.21 ± 8.66 (0.56)	6	5	0	0.12 – 0.56<br>0.25 ± 0.18 (0.17)
Harghita	46.36	25.80	60	0	Lu	-		-	-	2	1	0	0.00 – 0.43<br>0.22
Covasna	45.50	26.11	60	0	Lu	2	2	0	0.16 – 0.21<br>0.19	2	2	0	0.38 – 0.39<br>0.39
Brașov	45.79	25.28	50	0	Lu	2	1	0	0.00 – 0.38<br>0.19	2	2	0	0.31 – 0.66<br>0.49
Sibiu	45.87	24.23	40	0	Lu	3	3	1	0.04 – 1.74<br>0.70 ± 0.91 (0.32)	3	3	0	0.13 – 0.81<br>0.54 ± 0.36 (0.67)
Alba	46.13	23.53	30	–100	Lu	-	-	-	-	2	2	0	0.31 – 0.76<br>0.54
Cluj	46.43	23.32	30	–100	Ph	2	2	0	0.07 – 0.27<br>0.17	2	2	1	0.04 – 4.92<br>2.48
Hunedoara	45.78	22.93	40	–50	Lu	2	1	0	0.00 – 0.03<br>0.	2	2	1	0.22 – 1.43<br>0.82
**Transylvania**	**45.50 – 47.44**	**22.93 – 26.11**	**30 – 60**	**–100 – 0**		**32**	**27<br>84%**	**10<br>31%**	**0.00 – 21.84<br>2.23 ± 5.40 (0.38)**	**37**	**35<br>95%**	**9<br>24%**	**0.04 – 4.92<br>0.87 ± 0.99 (0.66)**
Buzău	45.27	26.77	30	–200	Ch	3	0	0	0.00	4	3	0	0.04 – 0.07<br>0.06 ± 0.02 (0.06)
Prahova	45.10	26.02	30	–100	Lu	1	0	0	0.00	5	4	0	0.06 – 0.50<br>0.19 ± 0.21 (0.10)
Dâmbovița	44.89	25.47	40	–100	Lu	5	0	0	0.00	10	10	0	0.06 – 0.93

													0.21 ± 0.27 (0.10)
Argeș	45.00	24.82	40	–100	Lu	14	5	0	0.00 – 0.39<br>0.08 ± 0.14 (0.00)	6	6	1	0.11 – 1.56<br>0.48 ± 0.57 (0.18)
Vâlcea	45.08	24.11	40	–100	Lu	5	3	0	0.00 – 0.19<br>0.09 ± 0.09 (0.08)	5	5	1	0.06 – 1.70<br>0.64 ± 0.69 (0.27)
Gorj	45.04	23.30	50	0	Lu	3	3	1	0.18 – 1.37<br>0.59 ± 0.68 (0.22)	2	2	0	0.26 – 0.85<br>0.56
Caraș-Severin	45.15	22.07	40	–50	Lu	3	2	0	0.00 – 0.40<br>0.14	3	2	1	0.03 – 1.53<br>0.72 ± 0.76 (0.61)
**Southern Hilly Area**	**44.89 – 45.27**	**22.07 – 26.77**	**30 – 50**	**–200 – 0**		**34**	**13<br>38%**	**1<br>3%**	**0.00 – 1.37<br>0.11 ± 0.25 (0.00)**	**35**	**32<br>91%**	**3<br>9%**	**0.03 – 1.70<br>0.38 ± 0.47 (0.17)**
Timiș	45.78	21.35	40	–200	Ch	29	17	6	0.00 – 5.63<br>0.88 ± 1.28 (0.11)	10	10	4	0.16 – 2.04<br>0.87 ± 0.58 (0.78)
Arad	46.10	21.08	30	–200	Ch	-		-	-	2	2	0	0.03 – 0.35<br>0.19
Bihor	47.07	21.92	30	–100	Lu	5	5	1	0.11 – 1.79<br>0.58 ± 0.70 (0.30)	5	4	1	0.18 – 1.41<br>0.62 ± 058 (0.46)
Satu-Mare	47.69	22.89	30	–150	Lu	3	2	0	0.00 – 0.45<br>0.23 ± 0.23 (0.25)	3	3	0	0.02 – 0.11<br>0.08 ± 0.05 (0.11)
**West Plain**	**45.78 – 47.69**	**21.08 – 22.89**	**30 – 40**	**–200 – –100**		**37**	**24<br>65%**	**7<br>19%**	**0.00 – 3.08<br>0.21 ± 0.58 (0.00)**	**20**	**19<br>95%**	**5<br>14%**	**0.02 – 2.04<br>0.62 ± 0.58 (0.43)**
Mehedinți	44.63	22.88	50	–100	Lu	1	0	0	0.00	2	2	0	0.28 – 0.29<br>0.28
Dolj	44.17	23.70	30	–200	Ch	4	3	0	0.00 – 0.20	4	1	0	0.00 – 0.58<br>0.14 ± 0.29 (0.00)
Olt	44.43	24.37	30	–200	Ph	4	3	0	0.00 – 0.21	4	4	0	0.06 – 0.54<br>0.30 ± 0.20 (0.31)
**Oltenia Plain**	**44.17 – 44.63**	**22.88 – 24.37**	**30 – 50**	**–200 – –100**		**9**	**6<br>67%**	**0<br>0%**	**0.00 – 0.21<br>0.10 ± 0.09 (0.11)**	**10**	**7<br>70%**	**0<br>0%**	**0.07 – 0.52<br>0.25 ± 0.18 (0.21)**
Botoșani	47.84	26.82	30	–100	Ch	1	0	0	0.00	2	2	0	0.06 – 0.11<br>0.09
Suceava	47.58	25.76	30	–100	Lu	2	1	0	0.00 – 0.22<br>0.11	2	2	0	0.03 – 0.20<br>0.11
Iași	47.25	27.31	30	–200	Ch	1	1	0	0.13	2	2	0	0.02 – 0.03<br>0.03
Neamț	46.97	26.40	30	–200	Lu	1	1	0	0.04	2	1	0	0.00 – 0.31<br>0.15

Vaslui	46.59	27.77	25	–250	Ch	2	1	0	0.00 – 0.03<br>0.02	2	2	0	0.02 – 0.10<br>0.06
Bacău	46.42	26.78	30	–200	Lu	3	1	0	0.00 – 0.05<br>0.02 ± 0.03 (0.00)	2	2	0	0.15 – 0.24<br>0.20
Galați	45.79	27.78	25	–300	Ch	3	0	0	0.00	3	3	0	0.03 – 0.16<br>0.09 ± 0.07 (0.08)
Vrancea	45.75	26.97	25	–200	Ch	1	0	0	0.00	2	0	0	0.00
**Moldavia**	**45.75 – 47.84**	**25.76 – 27.78**	**25 – 30**	**–300 – –100**		**14**	**5<br>36%**	**0<br>0%**	**0.00 – 0.22<br>0.11 ± 0.25 (0.00)**	**17**	**14<br>82%**	**0<br>0%**	**0.02 – 0.31<br>0.11 ± 0.09 (0.09)**
Brăila	45.11	27.68	25	–300	Ch	2	0	0	0.00	2	1	0	0.00 – 0.15<br>0.07
Ialomița	44.64	27.30	25	–300	Ch	1	0	0	0.00	2	2	0	0.07 – 0.41<br>0.24
Ilfov	44.61	26.12	25	–200	Lu	1	0	0	0.00	2	2	0	0.08 – 0.14<br>0.11
Teleorman	43.39	25.21	30	–250	Ch	1	1	0	0.08	2	1	0	0.00 – 0.31<br>0.16
Giurgiu	44.16	25.90	25	–200	Ch	1	0	0	0.00	2	2	0	0.15 – 0.85<br>0.50
Călărași	44.12	27.20	25	–300	Ch	1	0	0	0.00	4	2	0	0.11 – 0.52<br>0.31 ± 0.29 (0.31)
**Southern Plain**	**43.39 – 45.11**	**25.21 – 27.68**	**25 – 30**	**–300 – –200**		**7**	**1**	**0**	**0.00 – 0.08<br>0.01 ± 0.03 (0.00)**	**14**	**10**	**0**	**0.00 – 0.85<br>0.20 ± 0.25 (0.12)**
Tulcea	44.98	28.77	20	–350	Ch	1	0	0	0.00	2	2	0	0.08 – 0.15<br>0.11
Constanța	44.27	28.31	20	–400	Ch	1	0	0	0.00	2	0	0	0.00
**Dobrogea**	**44.27 – 44.98**	**28.31 – 28.77**	**20**	**–400 – –350**		**2**	**0**	**0**	**0.00**	**4**	**2**	**0**	**0.00 – 0.15<br>0.06 ± 0.07 (0.04)**
**ROMANIA**	**43.39 – 47.84**	**21.08 – 28.77**	**20 – 60**	**–400 – 0**		**135**	**76<br>56%**	**18<br>13%**	**0.00 – 21.84<br>0.45 ± 2.03 (0.00)**	**137**	**119<br>87%**	**17<br>12%**	**0.00 – 4.92<br>0.82 ± 0.98 (0.50)**

Climate type by agricultural region and aridity indices (Iar-dM and CWD) in 1900–2000: Humid climate in Transylvania; Humid-balanced climate in Southern Hilly Area; Sub-humid climate in Oltenia Plain and the West Plain; Semi-arid climate in Moldavia and the Southern Plain; Arid climate in Dobrogea [133].

Soil type (scale 1:1,500,000): Ch— chernozem; Lu— luvisol; Ph—phaeozem [11,12].

Maximum limit of *Fusarium*-damaged kernels in common wheat is 1% [78].

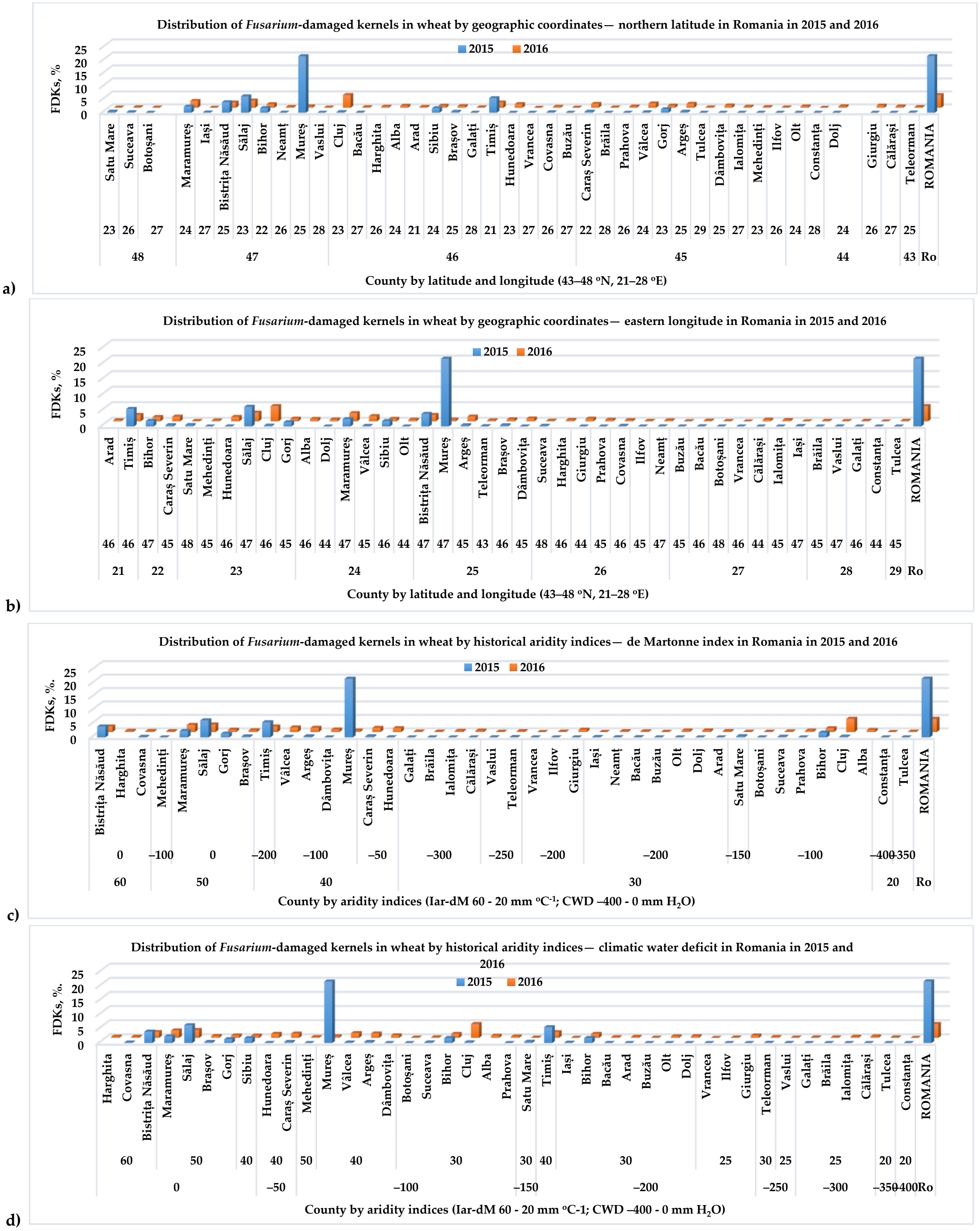

Figure S2. Spatial and geographic distribution of maximum values of *Fusarium*-damaged kernels (FDKs) in common wheat in Romania in 2015 and 2016, by: (**a**, **b**) geographic coordinates— Northern latitude, °N and Eastern longitude, °E; (**c**, **d**) historical aridity indices in 1900–2000— de Martonne aridity index, Iar-dM and climatic water deficit, CWD.

toxins

MDPI

Table S2. Maximum level of *Fusarium*-damaged kernels (FDKs) in common wheat by geographic position (agricultural region, geographic coordinates, county and locality), aridity indices (de Martonne, Iar-dM; climatic water deficit, CWD), hydrographic basin and wheat variety in Romania in 2015 and 2016.

Year	Agricultural Region	Geographic Coordinates		County	Aridity Indices, in 1900–2000		Locality	Soil	Hydrographic Basin	Common Wheat Variety	FDKs %, in 2015–2016	Precipitation, mm			Air Temperature, °C			Meteo Station
		Latitude, °N	Longitude, °E		Iar-dM, mm °C[-1]	CWD, mm H_2O						May	June	July	May	June	July	
2015				Bistrița-Năsăud, BN	60		Șieu-Măgheruș	Lu	Șieu x Dipșa	Andrada	1.14	113.8	84.4	41.6	15.7	19.2	21.7	Bistrița, BN
							Sîngeorzu-Nou		Someșu Mare	Arieșan	4.05							
		47	24	Maramureș, MM	50	0	Șomcuta Mare		Bârsău (trib. of Someș)		2.02	97.8	58.2	23.6	16.0	20.1	22.7	Baia-Mare, MS
							Odești, Baia Mare		Someș	Glosa	2.36							
	Transylvania		23	Sălaj, SJ			Năpradea *			Acrotos	1.68	132.2	48.4	38.4	16.0	20.1	22.9	Zalău, SJ
							Măieriște		Crasna	Potaisa	6.33							
		46		Mureș, MS	40	–100	Sâncraiu de Mureș	Lu-Ph	Mureș	Exotic	21.84	67.6	127.4	32.1	16.0	19.1	22.0	Tg, Mureș, MS
							Sânpetru de Câmpie				1.90							
		45	24	Sibiu, SB	40	0	Brăteiu	Lu	Târnava Mare	Eder Ron	1.74	85.8	183.6	27.4	15.2	18.0	21.0	Dumbrăveni, SB
	Southern Hilly Area	45	23	Gorj, GJ	50	0	Drăguțești	Lu	Jiu	Other	1.37	35.2	34.0	38.8	18.0	20.9	25.4	Tg. Jiu, GJ
		47		Bihor, BH	30	–100	Șimian	Lu	-	Exotic	1.79	79.0	34.9	24.2	16.7	20.5	24.1	Săcuieni, BH
	West Pain	45	21	Timiș, TM	40	–200	Lovrin	Ch	-	Crişana	5.63	71.3	6.5	95.5	17.6	21.4	25.0	Sânnicolaul Mare, TM
										Altigo	3.08							
							Sânicolaul Mare		-	Franz Iosef	1.34							
							Buziaș		Șurgani	Soisson	1.25	38.6	46.0	6.4	17.2	20.6	24.3	Lugoj, TM
							Comloșu Mare		-	Midas	1.79	71.3	6.5	95.5	17.6	21.4	25.0	Sânnicolaul Mare, TM
							Cărpiniș		-	Arnold	3.21							

Year	Agricultural Region	Geographic Coordinates: Latitude, °N	Geographic Coordinates: Longitude, °E	County	Aridity Indices, in 1900–2000: Iar-dM, mm °C^{-1}	Aridity Indices, in 1900–2000: CWD, mm H_2O	Locality	Soil	Hydrographic Basin	Common Wheat Variety	FDKs %, in 2015–2016	Precipitation, mm: May	Precipitation, mm: June	Precipitation, mm: July	Air Temperature, °C: May	Air Temperature, °C: June	Air Temperature, °C: July	Meteo Station
2016	Transylvania	47	24	Bistrița-Năsăud, BN	60	0	Bistrița-Năsăud	Lu	Bistrița	Arieșan	1.03	44.2	117.8	65.0	14.5	20.4	21.0	Bistrița, BN
		47	24							Andrada	1.68							
		47	24							Other	2.06							
		47	24	Maramureș, MM			Viile Apei, Șeini		Someș	Adesso	1.51	68.4	151.8	91.8	15.3	20.9	21.6	Baia Mare, MS
		47	24				Arduzel, Ulmeni			Arieșan	1.44							
		47	24				Baia-Mare		Săsar (affl. of Someș)	Adesso	2.65							
		47	23	Sălaj	50	0	Măeriște		Crasna	Arieșan	2.80	97.2	137.4	83.8	15.4	20.5	21.5	Zalău, SJ
		46	23	Cluj	30	−100	Apahida	Ch	-	Renan	4.92	84.2	113.6	113.4	13.9	19.7	20.4	Cluj Napoca, CJ
		45	22	Hunedoara	40	−50	Peșteana	Lu	-		1.43	99.0	208.2	87.6	15.1	20.5	21.6	Caransebeș, CS
	Southern Hilly Area	45	22	Caraș-Severin		−50	Măureni	Lu	-	Arezo	1.53	78.1	180.8	88.4	15.6	21.2	22.2	Lugoj, TM
		45	24	Vâlcea	40	−100	Diculești		Olteț (trib. of Olt)	Exotic	1.70	78.6	46.6	28.8	15.3	21.7	23.8	Drăgășani, VL
		45	24	Argeș			Albota		Teleorman (trib. of Vedea)	Trivale	1.56	93.0	123.8	14.4	14.7	21.2	23.0	Pitești, AG
	West Pain	47	21	Bihor	30	−100	Sânicolaul Roman	Lu	-	Florian	1.41	46.4	168.1	85.3	16.4	21.3	22.5	Oradea, BH
		45		Timiș	40	−200	Lovrin	Ch	-	Lovrin 34	1.13	52.7	218.5	52.9	16.5	21.6	23.0	Sânnicolaul Mare, TM
							Buziaș		Șurgani	Bekes	1.09	78.1	180.8	88.4	15.6	21.2	22.2	Lugoj, TM
							Ciacova		Timișul Mort	Samson	1.42							

Giarmata	-	Apache	2.04	51.2	177.8	76.3	16.3	21.6	22.9	Timișoara, TM

Climate type by agricultural region and aridity indices (Iar-dM and CWD) in 1900–2000: Humid climate in Transylvania; Humid-balanced climate in Southern Hilly Area; Sub-humid climate in Oltenia Plain and the West Plain; Semi-arid climate in Moldavia and the Southern Plain; Arid climate in Dobrogea [133].

Soil type (scale 1:1,500,000): Ch— chernozem; Lu— luvisol; Ph—phaeozem [11,12].

Hidrographic Basin: trib.— tributary; affl.— affluent. The rivers of the Transylvania and West Plains regions are Tisza river' affluents, a significant affluent of the Danube River. The rivers of the Southern Hilly Area, Oltenia Plain, the Southern Plain and Moldavia regions are affluents of the Danube River.

Maximum limit of *Fusarium*-damaged kernels in common wheat is 1% [78].

Table S3. *Fusarium*-damaged kernels (FDKs) in common wheat by geographic coordinates—northern latitude in Romania in 2015 and 2016.

Latitude, °N	*Fusarium*-Damaged Kernels (FDKs) in Common Wheat by Geographic Coordinates—Northern Latitude in Romania in 2015 and 2016							
	2015				2016			
	No. of Samples; Incidence			Interval of FDKs in Common Wheat, Mean ± SD (Median), %	No. of Samples; Incidence			Interval of FDKs in Common Wheat, Mean ± SD (Median), %
	Total	≥0.01%	≥1%		Total	≥0.01%	≥1%	
43	1	1<br>100%	0<br>0%	0.08	2	1<br>100%	0<br>0%	0.31
44	18	6<br>33.33%	0<br>0%	0.00 – 0.21<br>0.04 ± 0.08 (0.00)	26	22<br>84.62%	0<br>0%	0.00 – 0.93<br>0.43 ± 0.29 (0.41)
45	74	37<br>50%	8<br>10.81%	0.00 – 5.63<br>0.74 ± 1.51 (0.20)	55	55<br>100%	8<br>14.29%	0.00 – 2.04<br>0.81 ± 0.67 (0.66)
46	15	11<br>73.33%	2<br>13.33%	0.00 – 21.84<br>3.71 ± 8.88 (0.05)	20	20<br>100%	1<br>5%	0.10 – 4.92<br>1.05 ± 1.72 (0.43)
47	27	21<br>77.78%	8<br>29.63%	0.00 – 6.33<br>1.92 ± 2.27 (1.12)	34	34<br>100%	8<br>23.53%	0.03 – 2.80<br>1.17 ± 1.21 (0.03)
Romania	135	76<br>56.30%	18<br>13.33%	0.00 – 21.84<br>1.24 ± 3.70 (0.08)	137	137<br>100%	17<br>12.41%	0.00 – 4.92<br>0.82 ± 0.98 (0.50)

Table S4. *Fusarium*-damaged kernels (FDKs) in common wheat by geographic coordinates—eastern longitude in Romania in 2015 and 2016.

Longitude, °E	*Fusarium*-damaged kernels (FDKs) in Common Wheat by Geographic Coordinates—Eastern Longitude in Romania in 2015 and 2016							
	2015				2016			
	No. of Samples; Incidence			Interval of FDKs in Common Wheat, Mean ± SD (Median), %	No. of Samples; Incidence			Interval of FDKs in Common Wheat, Mean ± SD (Median), %
	Total	≥0.01%	≥1%		Total	≥0.01%	≥1%	
21	34	22<br>64.71%	7<br>20.59%	0.00 – 5.63<br>3.71 ± 2.72 (3.71)	17	17<br>100%	5<br>29.41%	0.35 – 2.04<br>1.27 ± 0.85 (1.41)
22	9	5<br>55.56%	0<br>0%	0.00 – 0.45<br>0.28 ± 0.25 (0.40)	10	10<br>100%	2<br>20%	0.11 – 1.43<br>0.64 ± 0.77 (0.29)
23	15	11<br>73.33%	3<br>20%	0.00 – 6.33<br>1.37 ± 2.48 (0.24)	15	15<br>100%	2<br>14.86%	0.58 – 4.92<br>1.89 ± 1.69 (1.14)
24	41	29<br>70.73%	8<br>19.51%	0.00 – 21.84<br>4.40 ± 7.82 (1.74)	35	35<br>100%	8<br>22.86%	0.54 – 2.65<br>1.41 ± 0.81 (1.56)
25	10	2<br>20%	0<br>0%	0.00 – 0.38<br>0.14 ± 0.16 (0.08)	20	20<br>100%	0<br>0%	0.20 – 0.93<br>0.56 ± 0.30 (0.55)
26	14	5<br>35.71%	0<br>0%	0.00 – 0.21<br>0.04 ± 0.08 (0.00)	21	21<br>100%	0<br>0%	0.00 – 0.50<br>0.22 ± 0.18 (0.14)

27	9	2<br>22.22%	0<br>0%	0.00 – 0.13<br>0.03 ± 0.05 (0.00)	11	11<br>100%	0<br>0%	0.03 – 0.52<br>0.23 ± 0.19 (0.16)
28	3	0<br>0%	0<br>0%	0.00	8	8<br>100%	0<br>0%	0.00 – 0.15<br>0.08 ± 0.11 (0.08)
Romania	135	76<br>56.30%	18<br>13.33%	0.00 – 21.84<br>1.24 ± 3.70 (0.08)	137	137<br>100%	17<br>12.41%	0.00 – 4.92<br>0.82 ± 0.98 (0.50)

Table S5. *Fusarium*-damaged kernels (FDKs) in common wheat by soil in Romania in 2015 and 2016.

Soil	*Fusarium*-Damaged Kernels (FDKs) in Common Wheat by Soil in Romania in 2015 and 2016							
	2015				2016			
	No. of Samples; Incidence			Interval of FDKs in Common Wheat, Mean ± SD (Median), %	No. of Samples; Incidence			Interval of FDKs in Common Wheat, Mean ± SD (Median), %
	Total	≥0.01%	≥1%		Total	≥0.01%	≥1%	
Chernozem	52	23<br>44.23%	6<br>11.54%	0.00 – 5.63<br>0.40 ± 1.45 (0.00)	46	46<br>100%	4<br>18.70%	0.00 – 2.04<br>0.36 ± 0.51 (0.16)
Phaeozem	8	8<br>100%	2<br>25%	0.21 – 21.84<br>7.44 ± 12.47 (0.27)	6	6<br>100%	1<br>16.66%	0.54 – 4.92<br>2.74 ± 2.52 (0.56)
Luvisol	75	45<br>60%	10<br>13.33%	0.00 – 6.33<br>0.95 ± 1.61 (0.22)	85	85<br>100%	12<br>14.25%	0.11 – 2.80<br>0.99 ± 0.80 (0.79)
Romania	135	76<br>56.30%	18<br>13.33%	0.00 – 21.84<br>1.24 ± 3.70 (0.08)	137	137<br>100%	17<br>12.41%	0.00 – 4.92<br>0.82 ± 0.98 (0.50)

Table S6. *Fusarium*-damaged kernels (FDKs) in common wheat by aridity— de Martonne aridity index in Romania in 2015 and 2016.

de Martonne Aridity Index (Iar-dM), mm °C^{-1} (1900–2000)	*Fusarium*-Damaged Kernels (FDKs) in Common Wheat by Aridity— de Martonne Aridity Index in Romania in 2015 and 2016							
	2015				2016			
	No. of Samples; Incidence			Interval of FDKs in Common Wheat, Mean ± SD (Median), %	No. of Samples; Incidence			Interval of FDKs in Common Wheat, Mean ± SD (Median), %
	Total	≥0.01%	≥1%		Total	≥0.01%	≥1%	
20	2	0<br>0%	0<br>0%	0.00 – 0.00<br>0.00	4	4<br>100%	0<br>0%	0.00 – 0.15<br>0.08 ± 0.11 (0.08)
25	14	2<br>14.29%	0<br>0%	0.00 – 0.03<br>0.00 ± 0.01 (0.00)	20	20<br>100%	0<br>0%	0.00 – 0.85<br>0.29 ± 0.28 (0.16)
30	30	19<br>63.33%	1<br>3.33%	0.00 – 1.79<br>0.25 ± 0.46 (0.11)	42	42<br>100%	2<br>4.76%	0.03 – 4.92<br>0.70 ± 1.22 (0.31)
40	69	39<br>56.52%	9<br>13.04%	0.00 – 21.84<br>3.78 ± 7.54 (0.40)	49	49<br>100%	8<br>16.33%	0.56 – 2.04<br>1.32 ± 0.50 (1.48)
50	13	10<br>76.92%	5<br>38.46%	0.00 – 6.33<br>2.09 ± 2.61 (1.37)	13	13<br>100%	4<br>30.77%	0.29 – 2.80<br>1.45 ± 1.14 (0.85)
60	7	6<br>85.71%	3<br>42.86%	0.21 – 4.05<br>2.13 ± 2.72 (2.13)	9	9<br>100%	3<br>33.33%	0.39 – 2.06<br>0.96 ± 0.95 (0.96)

Romania	135	76<br>56.30%	18<br>13.33%	0.00 – 21.84<br>1.24 ± 3.70 (0.08)	137	137<br>100%	17<br>12.41%	0.00 – 4.92<br>0.82 ± 0.98 (0.50)

De Martonne's aridity index (Iar-dM) = P/(T+10), where P is the mean annual precipitation (mm), and T is the mean annual air temperature (°C) [133,143].

Table S7. *Fusarium*-damaged kernels (FDKs) in common wheat by aridity— climatic water deficit in Romania in 2015 and 2016.

**Climatic Water Deficit (CWD), mm H_2O (1900–2000)**	***Fusarium*-Damaged Kernels (FDKs) in Common Wheat by Aridity— Climatic Water Deficit in Romania in 2015 and 2016**							
	**2015**				**2016**			
	**No. of Samples; Incidence**			**Interval of FDKs in Common Wheat, Mean ± SD (Median), %**	**No. of Samples; Incidence**			**Interval of FDKs in Common Wheat, Mean ± SD (Median), %**
	**Total**	**≥0.01%**	**≥1%**		**Total**	**≥0.01%**	**≥1%**	
–400	1	0<br>0%	0<br>0%	0.00	2	0<br>0%	0<br>0%	0.00
–350	1	0<br>0%	0<br>0%	0.00	2	2<br>100%	0<br>0%	0.08 – 0.15<br>0.12
–300	7	0<br>0%	0<br>0%	0.00	11	11<br>100%	0<br>0%	0.15 – 0.52<br>0.31 ± 0.18 (0.29)
–250	3	2<br>66.67%	0<br>0%	0.03 – 0.08<br>0.06 ± 0.04 (0.06)	4	4<br>100%	0<br>0%	0.10 – 0.31<br>0.21 ± 0.15 (0.21)
–200	48	26<br>54.17%	6<br>12.50%	0.00 – 5.63<br>0.63 ± 1.76 (0.05)	38	38<br>100%	4<br>10.53%	0.00 – 2.04<br>0.47 ± 0.58 (0.31)
–150	3	2<br>66.67%	0<br>0%	0.00 – 0.45	3	2<br>66.67%	0<br>0%	0.02 – 0.11<br>0.08 ± 0.05 (0.11)
–100	43	22<br>51.16%	3<br>7%	0.00 – 21.84<br>2.25 ± 6.52 (0.19)	47	47<br>100%	4<br>8.51%	0.11 – 4.92<br>1.18 ± 1.36 (0.76)
–50	5	3<br>60%	0<br>0%	0.03 – 0.40<br>0.22 ± 0.26 (0.22)	5	5<br>100%	2<br>40%	1.43 – 1.53<br>1.48 ± 0.07 (1.48)
0	24	21<br>87.50%	9<br>37.50%	0.21 – 6.33<br>2.35 ± 2.18 (2.36)	25	25<br>100%	7<br>28%	0.39 – 2.80<br>1.33 ± 1.01 (1.36)
Romania	135	76<br>56.30%	18<br>13.33%	0.00 – 21.84<br>1.24 ± 3.70 (0.08)	137	134<br>97.81%	17<br>12.41%	0.00 – 4.92<br>0.82 ± 0.98 (0.50)

Climatic Water Deficit (CWD) = P – ETo-PM, where P is the precipitation sum (mm), and ETo-PM is the Penman-Monteith reference evapotranspiration (mm) [133,134].

toxins

Table S8. Correlation between *Fusarium*-damaged kernels (FDKs) in common wheat and the agrometeorological factors, historical aridity indices and geographic coordinates in Romania in the dry year 2015 (Pearson correlation coefficient).

**Correlations in dry 2015**		***Fusarium*-damaged kernels (FDKs)**	**Agrometeorology, May–June 2015**		**Aridity Indices, 1900–2000**		**Geographic Coordinates**	
			**Precipitation**	**Air Temperature**	**de Martonne Aridity Index**	**Climatic Water Deficit**	**Latitude**	**Longitude**
Fusarium-damaged kernels (FDKs)	Pearson Correlation	1.000	0.259	–0.171	0.249	0.209	0.246	–0.215
	Significance (two-tailed)		0.102	0.286	0.117	0.190	0.121	0.176
	N	41	41	41	41	41	41	41
de Martonne Aridity Index (Iar-dM)	Pearson Correlation	0.249	0.589 ***	–0.623 ***	1.000	0.832 ***	0.231	–0.420 **
	Significance (two-tailed)	0.117	0.000	0.000		0.000	0.147	0.006
	N	41	41	41	41	41	41	41
Climatic Water Deficit (CWD)	Pearson Correlation	0.209	0.616 ***	–0.667 ***	0.832 ***	1.000	0.421 **	–0.555 ***
	Significance (two-tailed)	0.190	0.000	0.000	0.000		0.006	0.000
	N	41	41	41	41	41	41	41
Latitude	Pearson correlation	0.246	0.114	–0.499 ***	0.231	0.421 **	1.000	–0.121
	Significance (two-tailed)	0.121	0.479	0.001	0.147	0.006		0.450
	N	41	41	41	41	41	41	41
Longitude	Pearson Correlation	–0.215	–0.502 ***	0.178	–0.420 **	0.555 ***	–0.121	1.000
	Significance (two-tailed)	0.176	0.001	0.265	0.006	0.000	0.450	
	N	41	41	41	41	41	41	41

*—correlation at the 0.05 level (two-tailed); **—correlation is significant at the 0.01 level (two-tailed); ***—correlation is very significant at the 0.001 level (two-tailed).

Table S9. Correlation between *Fusarium*-damaged kernels (FDKs) in common wheat and the agrometeorological factors, aridity indices and geographic coordinates in Romania in the rainy year 2016 (Pearson correlation coefficient).

**Correlations in rainy 2016**		***Fusarium*-damaged kernels (FDKs)**	**Agrometeorology, May–June 2016**		**Aridity Indices, 1900–2000**		**Geographic Coordinates**	
			**Precipitation**	**Air Temperature**	**de Martonne Aridity Index**	**Climatic Water Deficit**	**Latitude**	**Longitude**
Fusarium-damaged kernels (FDKs)	Pearson Correlation	1.000	0.290	–0.217	0.367 *	0.457 **	0.214	–0.526 ***
	Significance (two-tailed)		0.066	0.172	0.018	0.003	0.179	0.000
	N	41	41	41	41	41	41	41
de Martonne Aridity Index (Iar-dM)	Pearson Correlation	0.367 **	0.287	–0.608 ***	1.000	0.832 ***	0.231	0.420 **
	Significance (two-tailed)	0.018	0.069	0.000		0.000	0.147	0.006
	N	41	41	41	41	41	41	41
Climatic Water Deficit (CWD)	Pearson Correlation	0.457 **	0.368 *	–0.688 ***	0.832 ***	1.000	0.421 **	–0.555 ***
	Significance (two-tailed)	0.003	0.018	0.000	0.000		0.006	0.000
	N	41	41	41	41	41	41	41
Latitude	Pearson correlation	0.214	0.291	–0.471 **	0.231	0.421 **	1.000	–0.121
	Significance (two-tailed)	0.179	0.065	0.002	0.147	0.006		0.450
	N	41	41	41	41	41	41	41
Longitude	Pearson Correlation	–0.526 ***	–0.310 *	0.161	–0.420 **	0.555 ***	–0.121	1.000
	Significance (two-tailed)	0.000	0.049	0.314	0.006	0.000	0.450	
	N	41	41	41	41	41	41	41

*—correlation at the 0.05 level (two-tailed); **—correlation is significant at the 0.01 level (two-tailed); ***—correlation is very significant at the 0.001 level (two-tailed).

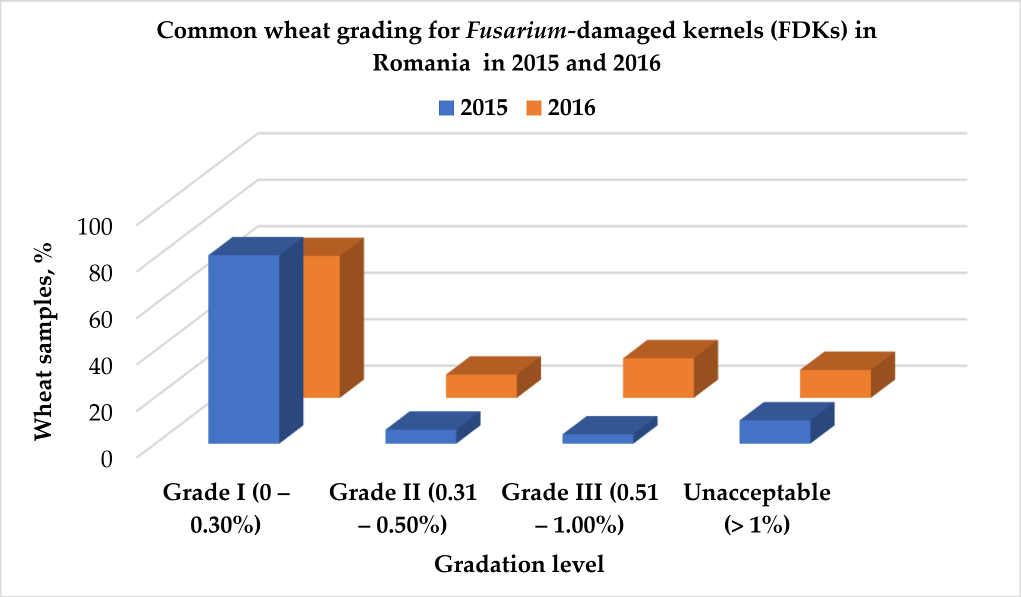

Figure S3. Common wheat grading for *Fusarium*-damaged kernels (FDKs) in Romania in the dry 2015 and the rainy 2016 year, respectively. The National Grading Plan for wheat consumption includes three levels: grade I – max. 0.3% (very good); grade II – max. 0.5% (good), grade III –- max. 1% (acceptable), and unacceptable – >1% (unacceptable for consumption) [78].
